# Economic Recovery but Stagnating Mental Health During a Global Pandemic? Evidence from Ghana and South Africa

**DOI:** 10.1111/roiw.12587

**Published:** 2022-05-02

**Authors:** Kathrin Durizzo, Edward Asiedu, Antoinette van der Merwe, Isabel Günther

**Affiliations:** ^1^ ETH Zürich Zurich Switzerland; ^2^ University of Ghana Business School Accra Ghana; ^3^ University of Passau Passau Germany

**Keywords:** COVID‐19, economic recovery, mental health, urban poor

## Abstract

Ghana and South Africa proactively implemented lockdowns very early in the pandemic. We analyze a three‐wave panel of households in Accra and Greater Johannesburg to study the mental and economic well‐being of the urban poor between the COVID‐19 lockdown and the “new normal” one year later. We find that even if economic well‐being has mostly recovered, life satisfaction has only improved slightly and feelings of depression are again at lockdown levels one year into the pandemic. While economic factors are strongly correlated with mental health and explain the differences in mental health between South Africa and Ghana, increasing worries about the future and limited knowledge about the pandemic (both countries) as well as deteriorating physical health (South Africa) and trust in government (Ghana) explain why mental health has not recovered. Therefore, we need broad and country‐specific policies, beyond financial support, to accelerate the post‐pandemic recovery of the urban poor.

## 1 Introduction

In the early phases of the COVID‐19 pandemic, many were concerned about the virus's impact in cities of low‐ and middle‐income countries (LMICs), given poor sanitation conditions, densely populated areas, compromised immunities, and underfinanced healthcare systems. The pressure that COVID‐19 outbreaks placed on countries with well‐financed healthcare systems early in the pandemic raised doubts about whether medical infrastructure in LMICs would be able to manage a rapid surge of infections. Due to these concerns, many governments of LMICs, such as Ghana and South Africa, were quicker to implement public lockdown regulations than high‐income countries (HICs) in Northern America and Europe. While government measures slowed down the reproduction of the virus and minimized mortality rates (Flaxman et al., 2020; Hsiang et al., 2020), the policies also imposed high economic costs on LMICs because of the large share of the population working in the informal economy, low incomes, and limited social security. Studies of LMICs during lockdowns clearly indicate that earnings decreased, and unemployment and food insecurity increased (Carsi Kuhangana et al., 2020; Hamadani et al., 2020; Jain et al., 2020; IPA, 2020; Warren et al., 2020a, 2020b; Stein et al., 2020; Durizzo et al., 2021; Egger et al., 2021; Kesar et al., 2021; Mahmud and Riley, 2021; Meyer et al., 2021).

A year and a half after the WHO declared COVID‐19 a pandemic in March 2020, many economies showed fast macro‐economic recovery. However, recovery for LMICs, especially in Africa, has been slower (IMF, 2021) and is expected to remain fragile due to the slow global distribution of vaccines (United Nations, 2021).

However, few longitudinal micro‐economic studies focus on the economic recovery of poor populations in LMICs from early in the pandemic to after the lifting of lockdown regulations. Using phone surveys in Ghana, four months after the regional lockdowns were lifted, Schotte et al. (2021) find evidence of a national decline in income for informal and small business owners. Using financial diaries taken from October 2019 to September 2020, Rönkkö et al. (2021) find that although household income in Bangladesh recovered after economies re‐opened, it was still below pre‐pandemic levels four months after the lockdown was lifted. Similarly, results from ongoing high‐frequency phone surveys in Burkina Faso, Ethiopia, Malawi, Mali, Nigeria, and Uganda reveal that although a substantial proportion of respondents returned to work, this has not necessarily translated to a full recovery in incomes (World Bank, 2021). In contrast, Innovation for Poverty Action, which conducted surveys in various countries, in May, August, and November 2020, show that employment fell substantially during the lockdown (February to May 2020), but recovered exceeding previous levels, in November 2020 (IPA, 2020). This positive trend can also be seen in hours worked, earnings, and food security, especially for the formally employed.

Given the different pace of economic recovery, further evidence is needed for LMICs to understand how economic factors have developed at the micro‐level from early in the pandemic to after public lockdowns. Moreover, at least to our knowledge, no study has followed the economic well‐being of households in LMICs up to one year after the start of the pandemic, entering a “new normal” in 2021 and no study has linked this longer‐term economic recovery to mental health in LMICs. Previous research on economic downturns linked to the global financial crises in 2008 has indicated that economic downturns can be associated with an increase in poor mental health and that this negative effect might remain even after the economy recovers due to job insecurity (for Germany: Avdic et al., 2020; for Australia: Black et al., 2022).

Moreover, apart from adverse economic impacts, COVID‐19 and the measures to slow the virus's reproduction has influenced many other factors that potentially have adverse mental health implications. For example, health‐related anxieties, such as the fear of personal and family contamination, could worsen mental health (Perrin et al., 2009; Pfefferbaum and North, 2020; Banks et al., 2021). Limited knowledge regarding the pandemic (Bäuerle et al., 2020), reduced mobility (Burdett et al., 2021), and limited social interactions (Pancani et al., 2021) could also have a negative effect on mental health. Other concerns include a significant increase in domestic abuse and violence during strict national lockdowns (Peterman et al., 2020; Banks et al., 2021) and overburdening guardians due to school closures (Sadique et al., 2008; Pierce et al., 2020; Shevlin et al., 2020). In addition, given the significant political challenges related to managing a pandemic, political uncertainty and public distrust in the government has the potential to exacerbate stress (Perrin et al., 2009; Bäuerle et al., 2020; Olagoke et al., 2020).

The impact of the consequences of COVID‐19 on mental health has, however, only been prominently analyzed and discussed for HICs. For example, using monthly online surveys from April 2020 to June 2021, Matsubayashi et al. (2022) find that adults in Japan were more likely to report symptoms related to anxiety and depression when they experienced a major change in employment or working conditions during COVID‐19. Cheng et al. (2020) find that life satisfaction in Singapore declined considerably when the nationwide lockdown was introduced, especially for households that reported a decline in income. Similarly, Clark and Lepinteur (2021) find for several European countries that the policy stringency is negatively associated with life satisfaction. Zajacova et al. (2020) show that mental health in Canada decreased considerably and that it is strongly associated with economic concerns. Banks et al. (2021) find that in various countries in Europe and the United States, COVID‐19 initially had a large negative impact on mental health. Despite some evidence of mental health recovery months later, not all mental health indicators have fully recovered.

The impact of COVID‐19 on mental health has been less frequently analyzed in LMICs, and if so, “only” during lockdowns of public life early in the pandemic and not after most measures have been lifted. Posel et al. (2021) use longitudinal data from rapid mobile phone surveys in South Africa to show that mental health was related to job loss; those who lost their job during the lockdowns were around 5 percent more likely to report depressive symptoms. A longitudinal study from Uganda, with in‐person surveys before the first lockdown and follow‐up phone surveys during the lockdown, finds that life satisfaction significantly decreased by 25 percent; one of the reasons being an increase in reported intimate partner violence (Mahmud and Riley, 2021). In Ethiopia, Meyer et al. (2021) find that 24 percent of women showed signs of depressive disorders when the government issued stay‐at‐home orders. Durizzo et al. (2021) find that 37 percent of the urban poor in Ghana and 51 percent of the urban poor in South Africa reported feeling down, depressed, and/or hopeless during public lockdowns in the spring of 2020. Conducting phone surveys from August to October 2020, Porter et al. (2021) find that reported symptoms of anxiety ranged widely from 41 percent in Peru, 18 percent in Ethiopia, 11 percent in India, to 9 percent in Viet Nam. Similar results were reported by Goularte et al. (2021) for Brazil, Wang et al. (2021) for seven Asian countries, Shamoon et al. (2020) for Pakistan and Cénat et al. (2021) for Haiti, DRC, Rwanda, and Togo.

Since most of these studies in both LMICS and HICS are cross‐sectional and were conducted when strict social distancing measures were in place, it is unclear how mental health changed throughout the pandemic, especially after strict lockdowns were lifted.

In this paper, we analyze poor urban households' mental and economic well‐being as they started to re‐enter public life with the global pandemic still ongoing. We interviewed about 1,000 poor urban households living in Accra, Ghana, and the Greater Johannesburg area, South Africa. We contacted respondents thrice: first, early in the lockdowns in April 2020, second, about four months later in August 2020, when regulations had been substantially relaxed in Ghana and to some extent also in South Africa; and third, almost one year later in March 2021 when all social distancing regulations were lifted and only hygienic measures were still in place, and vaccine campaigns had begun in Ghana and South Africa.

In doing so, we make three important contributions to our understanding of the impact of the pandemic on the urban poor's well‐being. First, the results will contribute to the small literature on the relationship between a global pandemic and mental health for LMICs. Second, we aim to identify the drivers of mental health during a global pandemic between two different countries and across time. Third, the results contribute to the few ongoing longer‐term economic recovery studies after public lockdowns, but during an ongoing pandemic. This is particularly important because of the different paces of economic recovery and the varying developments in mental health discussed in the literature.

We compare South Africa with Ghana, because on the one hand these are both middle‐income countries in Africa (a context that has been studied less when it comes to economic and mental health recovery), had some of the highest numbers of COVID‐19 cases in Africa, experienced a similar timing of the two COVID‐19 infection waves, and started COVID‐19 vaccine campaigns at similar times. On the other hand, the two country contexts differ considerably in terms of the duration of strict government measures to limit social interactions at the beginning of the pandemic. Moreover, the economic situation of the urban poor in each country also differs substantially, with many South African urban poor unemployed and many Ghanaian urban poor self‐employed. We focus on one of the most vulnerable groups, the population living in low‐income settlements in major cities, where public lockdowns are expected to have more severe impacts. Our results could help design policies to assist vulnerable communities in recovery after moving out of strict lockdowns.

We find that even if economic well‐being has mostly recovered after a year, life satisfaction has improved only slightly and feelings of depression are again at lockdown levels one year into the pandemic, after some improvements shortly after the first lockdown. Moreover, mental health indicators are, in general, worse among the urban poor in South Africa than in Ghana. Our results indicate that while economic factors are strongly correlated with mental health and explain the differences in mental health between South Africa and Ghana, increasing worries about future income and deteriorating knowledge about the pandemic explain why mental health has not recovered one year after the lockdown, while lack of social interactions seems to be less important. Nevertheless, key country differences exist: economic factors, trust in the government, and domestic violence seem to be more important for mental health in Ghana, whereas health factors seem to be more important in South Africa. Therefore, also for the poorest, we need to consider broader and country‐specific policies beyond financial support to accelerate the post‐pandemic recovery of mental health.

## 2 Context for Ghana and South Africa

**Figure 1:**
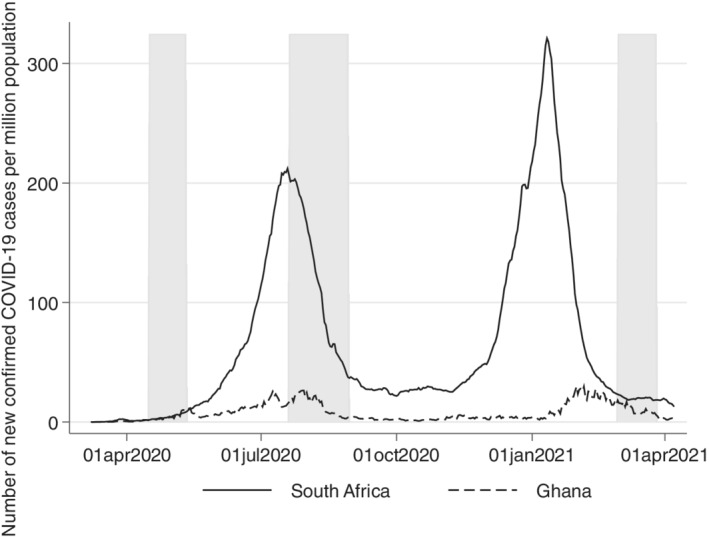
Number of total confirmed COVID‐19 cases per million population in South Africa and Ghana since March 1, 2020. *Notes*: A 7‐day moving average was calculated to display the number of daily cases. The gray shading indicates when the survey took place for both countries. *Source*: John Hopkins University (2021)—last update April 08, 2021

At the time of writing, Africa has reported 8.65 million confirmed COVID‐19 cases and 222,881 deaths (November 30, 2021; Johns Hopkins University, 2021). South Africa and Ghana, both middle‐income countries, have some of the highest numbers of total confirmed cases in Africa, with 2,968,052 infections and 89,843 deaths and 130,920 infections and 1,209 deaths, respectively (November 30, 2021; Johns Hopkins University, 2021). Within the study duration, April 2020 until March 2021, South Africa and Ghana had two COVID‐19 infection waves: the first peak was around July and August 2020 and the second in January and February 2021 (see Figure 1). Overall, South Africa was more adversely affected than Ghana, with about eight times more cases per million people during the peak. Both countries experienced third waves that exceeded the timeline of our study, lasting until around July 2021 in South Africa and August 2021 in Ghana (November 30, 2021; Johns Hopkins University, 2021).

In February and March 2021, at the end of our study period, South Africa and Ghana were some of the first countries in Africa to implement COVID‐19 vaccine distribution campaigns (South African Government, 2021; WHO, 2021). However, vaccination coverage remains low, mainly due to global shortages in vaccine supply. As of November 2021, 35 percent of South Africans and 11 percent of Ghanaians, respectively, received at least one dose of COVID‐19 vaccines (WHO, 2021).

**Figure 2:**
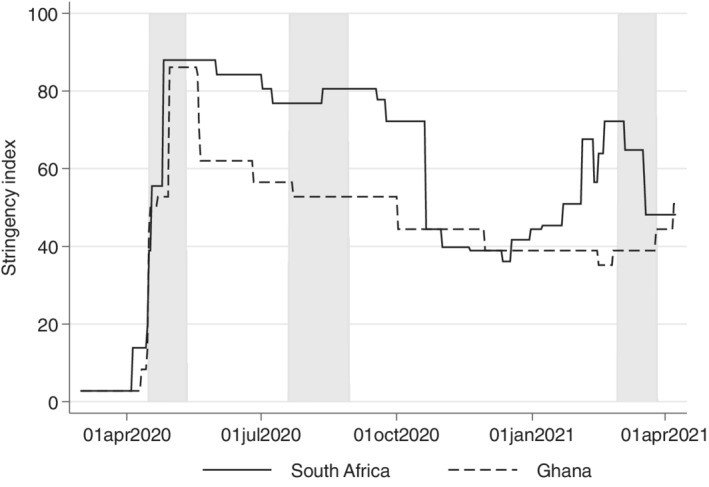
Stringency index from South Africa and Ghana since March 1, 2020. *Notes*: The Government Response Stringency Index is a composite measure based on nine response indicators, including school closures, workplace closures, and travel bans, rescaled to a value from 0 to 100 (100 = strictest response). This index should not be interpreted as “scoring” the appropriateness or effectiveness of a country's response; it simply records the number and the strictness of government policies. The gray shading indicates when the survey took place for both countries. *Source*: Hale et al. (2021)—last updated April 8, 2021

Although HICs, on average, reported their first wave much earlier, have experienced more waves, and have higher confirmed cases than in LMICs^1^ (November 30, 2021; Johns Hopkins University, 2021), HICs were slower to implement social distancing regulations and have mostly implemented less stringent measures throughout the pandemic. In contrast to many HICs, but similar to many other LMICs, both Ghana and South Africa implemented strict lockdowns of public life to limit the spread of COVID‐19 even before the 100th confirmed case (see Figure 2). South Africa even implemented one of the strictest lockdowns globally and only gradually lifted the restrictions (SACoronavirus, 2020), but by October 2020, most restrictions — except a small number of regulations such as mandatory mask usage and some limitations on large gatherings — had been eased (GardaWorld, 2020). In contrast, due to fears about worsening economic conditions (Akinwotu and Asiedu, 2020), Ghana lifted the public lockdown in the most affected cities (Accra and Kumasi) after only three weeks (see Figure 2).

We contacted respondents thrice between April 2020 and March 2021. First, in April 2020, when COVID‐19 cases were still relatively low (see Figure 1), but when strict lockdowns were implemented in both counties (see Figure 2). In both of our sample areas, only essential services were allowed to be open, but schools were closed. Additionally, in South Africa, the sale of alcohol and tobacco products was prohibited.

The second survey was conducted in August 2020, shortly after the peak of the first wave, when most restrictions had been eased in Ghana. However, schools only re‐opened for final‐year university students and senior and junior high school students to allow them to write their final exams (Presidency Republic of Ghana, 2020) — all other school levels remained closed. In contrast to Ghana, few restrictions on public life had been lifted in South Africa by the second survey round in August 2020 (see Figures 1 and 2). South Africans still had to remain at home except when going to work or school, purchasing goods, or exercising outside during the day (SACoronavirus, 2020).

The last survey round was conducted in March 2021 after the second COVID‐19 wave (see Figure 1), when COVID‐19 vaccine campaigns had started and when hygienic measures were the only COVID‐related measures remaining in effect in both countries (see Figure 2). All businesses, including the sale of alcohol and tobacco in South Africa, were operational. All land borders and airports were open, and all schools from the primary level to the university level were fully re‐opened. Nevertheless, sanitary measures and mask usage were still enforced in both countries (SACoronavirus, 2020; Presidency Republic of Ghana, 2021).

As a result, respondents in both countries completed surveys at similar times regarding COVID‐19 infections (before the first wave, during the first wave, and after the second wave) and when COVID‐19 vaccines were introduced (shorty before the third survey round). While South Africa had a longer public lockdown, Ghana's schools were closed longer. Given these similarities and differences, it is particularly interesting to compare the economic and mental health recovery in these two countries.

## 3 Methodology and Data

### 3.1 Data

We conducted three rounds of fully structured phone surveys of households living in 18 urban low‐income settlements in the Greater Accra region in Ghana^2^ and two municipalities in the Greater Johannesburg area^3^ (see Figures A1 and A2 in the Appendix).^4^ Since the first survey round was conducted during strict lockdowns of public life, phone surveys were the only possibility to reach out to low‐income urban respondents. Compared to online or SMS surveys, phone surveys allowed the research team to survey vulnerable respondents who were illiterate or without internet. We obtained ethical approval for this research from universities in Ghana, South Africa and Switzerland (available from the authors upon request). While the baseline survey took place during the lockdown (April 2020), the follow‐up surveys were conducted around four months (August 2020) and again approximately 11 months after the baseline survey (March 2021).

Three enumerators in South Africa and 16 enumerators in Ghana conducted the survey with the same person in the household in each of the three survey rounds. All enumerators had previous experience in conducting phone surveys and were trained by one of the co‐authors for this study. The questionnaire included questions on participants' age, gender, living conditions, economic situation, food consumption, stress factors, knowledge and perception of COVID‐19 and vaccinations, and life satisfaction and feelings of depression. The Ghanaian questionnaire included a few additional questions and took on average 38 min to complete, compared to 25 min in South Africa (the questionnaires for both countries are available upon request).

The sample in Ghana was a representative sample provided by the Ghana Statistical Service, which was randomly drawn from the most recent Ghana Living Standard Survey (GLSS7) carried out in 2017 from the 18 poorest settlements in the capital city Accra according to GLSS (stratified at and proportional to the settlement level). From the 1,034 persons who answered the first survey round in Ghana, 8 percent did not want to be called again. Of the numbers we could contact again, 14 percent were not valid or unanswered, and 5 percent of respondents did not want to participate in the survey again. We excluded 15 respondents who were from the same household, but were not the same person interviewed in the first survey round. In total, 736 respondents answered all three survey rounds in Ghana.

The sample in South Africa was drawn from the database of a study conducted in 2013 that used randomly selected households in two urban settlements of Ekurhuleni of Johannesburg (Nova Institute, 2016). A clustered sampling approach in combination with geographic sampling at the suburb level was used.^5^ The two South African townships east of Johannesburg belong to a larger municipality, Ekurhuleni, one of the three municipalities with the highest estimated share of people living in poverty in the country (Statistics South Africa, 2020). In South Africa, of the 409 persons who answered the first round of the survey, 10 percent did not want to be called back, 37 percent could not be reached due to invalid phone numbers or non‐responsive calls, and 18 percent did not want to participate when called a second time. We excluded 2 percent because they were not the same person surveyed in the first round. In total, 128 respondents answered all three survey rounds in South Africa.

Response rates for the second round in Ghana (83 percent) and South Africa (67 percent) are similar to follow‐up phone surveys in other LMICs (IPA, 2020; World Bank, 2021). However, the South African response rate dropped steeply in the third round, to 30 percent of the baseline. A potential reason for the drop could be that spam calls had massively increased in South Africa since mid‐2020 and people might have become less likely to pick up the phone if they did not recognize the phone number (Kok, 2020). In Ghana, the response rate only decreased slightly to 71 percent of the original sample. To address potential attrition bias, the drivers of attrition between the baseline and the second and third round samples on respondent demographics were estimated for both countries using a logistic regression model (see Table A1 in the Appendix). Testing for 22 covariates, the results show that in South Africa younger people and households with private toilets left the sample at a higher rate than the rest of the sample between the first and the third (final) survey rounds. For Ghana, we find that women and households not depending on national grants were more likely to leave the sample. To account for this potential bias in the sample, we used inverse probability weighting (IPW) for all analyses in this paper (Tables 1, 2, 3), reweighting all observations based on Table A1, and dropping all observations that were not interviewed in all three rounds, i.e., using a balanced reweighted sample (Wooldridge, 2010). Results do not change when IPW is not used (results available from the authors upon request).

### 3.2 Sample Description

Our sample highlights the situation experienced by urban households living in low‐income settlements in the Greater Accra region in Ghana and the Greater Johannesburg area in South Africa. Both study areas are among the poorest urban settlements in the two countries. However, the Ghanaian sample has proportionally fewer women (35 percent) than the South African one (76 percent; see Table A2 in the Appendix). In the case of Ghana, telephone numbers were randomly drawn from the Ghana Living Standard Survey (GLSS7), which listed information on the household heads, who are primarily male. In the South African sample, contact details were sourced from a previous research project on indoor smoke pollution (Nova Institute, 2016). During this project, the South African partner visited a random subset of households from the areas, and women were more likely to be at home. We thus control for the respondent's gender in all our analyses (Tables 2 and 3) to make sure our results are not driven by gender. We also replicated all descriptive statistics (Table 1 and Figure A3) and regressions (Tables 2 and 3) separately for men and women (see Appendix, Tables A4–A8 and Figure A4). The described trends in Sections 4.1 and 4.2 are not markedly different for men or women in either country.

More Ghanaian respondents have at least completed primary education (92 percent vs. 75 percent for South Africa), fewer Ghanaians were unemployed before the lockdown (4 percent vs. 58 percent for South Africa), and more Ghanaians were self‐employed before the lockdown (39 percent vs. 4 percent for South Africa). Moreover, in South Africa, national grants constitute a major share (65 percent) of households' income, whereas in Ghana, income from own businesses (59 percent) or employment (22 percent) are mentioned most often (see Table A2 in the Appendix). Work in South Africa is relatively more formalized; out of the 16 percent of South Africans who are employed, 55 percent have a contract. In Ghana, of the 43 percent participants who are employed, 25 percent have a contract. Therefore, despite both samples representing poor urban populations in middle‐income countries, the economic characteristics of the urban poor are quite different in the two countries, which makes for an interesting comparison.

### 3.3 Methodology

Our analysis focuses on two self‐reported mental health outcomes: life satisfaction and feeling depressed. To identify respondents' subjective overall well‐being, life satisfaction is measured on a scale from zero to 10 with the question “How satisfied are you with your life at the moment?” In the literature, life satisfaction is used as a first indicator to identify vulnerable groups at risk for depression (Rissanen et al., 2011; Gigantesco et al., 2019). It is shown in the literature that the evaluation of life satisfaction can change considerably depending on what areas of life the person focuses on (e.g., overall life satisfaction vs. life satisfaction in terms of health, see Cheng et al., 2020). Moreover, life satisfaction is a long‐term indicator and might not change quickly. Additionally, since life satisfaction can be defined as an individual's evaluation of their life as a whole based on their personal goals and achievements, but conditional on their environment, people might adjust their goals in times of shocks and therefore report their life satisfaction based on their “new” normal (Rissanen et al., 2011; Gigantesco et al., 2019).

To include a more specific and more time‐variant indicator of mental health in our analysis, we also consider a second mental health outcome: feeling down, depressed, and hopeless. Feeling depressed is measured on a five‐point Likert scale (“strongly applies” to “does not apply at all”). Although this is a rudimentary indicator of depression that might be less accurate than psychological diagnostic test questions, such as the nine‐point Patient Health Questionnaire (PHQ‐9) or seven‐point Generalized Anxiety Disorder (GAD‐7; e.g., Shevlin et al., 2020; O'Connor et al., 2021), it is in line with assessments of depression used in other studies (Witteveen and Velthorst, 2020; Mahmud and Riley, 2021). Moreover, such comprehensive diagnostic tests would have been difficult or even impossible to conduct using mobile phone data collection because attention span decreases much faster during phone surveys than during in‐person surveys.

The analysis is done in two steps. First, we analyze how mental health and economic factors have evolved since the start of the pandemic (Section 4.1) and how other factors either directly linked to the pandemic (i.e., knowledge about it) or linked to public movement restrictions during the lockdown (i.e., lower income) have evolved over time (Section 4.2). In a second step (Section 4.3), we investigate the impact of personal characteristics, such as gender, age, number of rooms per person, household with children, shared water source, shared toilet, education, and national grants as primary income before the lockdown, as well as a set of time‐variant economic factors, such as job security, financial liquidity, and food security on mental health. In addition, we analyze both the impact of public lockdown stress factors (such as social interactions, trust in government, worries about future income, or food^6^) and the impact of pandemic stress factors (such as physical health, knowledge about the pandemic, or worries about the health of the family) on mental health. We are also interested in understanding which of these factors explain observed differences in mental health between countries and across time.

To explore time‐variant and time‐invariant effects simultaneously, we first use a pooled OLS regression. The time‐invariant personal characteristics are denoted by ($X_{i}$), the time‐variant economic factors as ($E_{i}$), lockdown stress factors as ($L_{i}$), and pandemic stress factors as ($P_{i}$). Survey rounds are denoted as ($T_{t}$), and country fixed effects as ($C_{i}$). We run the regression separately for both outcome variables ($Y_{i,t}$): life satisfaction (scale 0–10) and feeling down, depressed, hopeless (scale 1–5).

(1) $$Y_{i,t}=\beta_{o}+\beta_{1}X_{i}+\beta_{2}E_{i,t}+\beta_{3}L_{i,t}+\beta_{4}P_{i,t}+\beta_{5}T_{t}+\beta_{6}C_{i}+\varepsilon_{i,t}$$

We introduce the independent variables with a stepwise approach (see Table 2) to test their explanatory power (*R* ^2^) and how much of the difference in mental health between countries and changes over time they explain. Moreover, we first estimate the regression for the entire sample (Table 2) and then separately for each country (Table A6 in the Appendix) to identify differences in drivers between countries. Regression coefficients indicate the association of a given variable with the person's life satisfaction or feelings of depression. A positive coefficient for life satisfaction means higher life satisfaction, whereas a positive coefficient for feelings of depression means higher feelings of depression. Therefore, we generally expect that covariates usually show the opposite sign for life satisfaction and feelings of depression.

Third (Section 4.3), we use the panel dimension of our sample by estimating a fixed effects model (with individual fixed effects $\left(I_{i}\right))$. The advantage of this model is that all unobserved time‐invariant heterogeneities across individuals, such as pre‐existing health risk factors that we have no data on, will be controlled for. However, this model does not allow us to analyze time‐invariant personal characteristics or general differences across countries (see equation 2). Again, the following regression was first estimated for the entire sample and then separately for each country to identify differences in drivers between countries (Table 3):

(2) $$Y_{i,t}=\beta_{o}+\beta_{1}X_{i}+\beta_{2}E_{i,t}+\beta_{3}L_{i,t}+\beta_{4}P_{i,t}+\beta_{5}T_{t}+\beta_{6}I_{i}+\varepsilon_{i,t}$$

Since participants stated if they felt depressed on a Likert scale, we also considered an ordered logit model for both equations (1) and (2) as a robustness check (see Appendix Tables A7 and A9). If not highlighted in the text, results do not change in comparison to the OLS regression. Moreover, we also run the two models (equations 1 and 2) controlling for a country stringency index (see Figure 2) and daily COVID‐19 cases per 1,000,000 people (see Figure 1). Results are available from the authors upon request. Since both variables are highly correlated with the survey rounds and do not vary much within one survey round for each country, the results are statistically insignificant.

## 4 Results

### 4.1 Mental Health Crisis Despite Recovery in Economic Well‐Being

In our sample from poor urban South Africa, respondents' average life satisfaction improved gradually over time from a low average of 3.9 during lockdown to 4.7 in August 2020 to 5.3 in March 2021 (scale is from zero to 10, see Table 1). In contrast, in our sample of low‐income settings in Greater Accra in Ghana, respondents' average life satisfaction has not changed much and even slightly decreased during the third wave, but always stayed above 5 from the lockdown in April 2020 to March 2021. Given our data, we unfortunately cannot determine if the recovery has reached pre‐pandemic levels in either country. The development of feeling down, depressed or hopeless—is a short‐term indicator of mental health in comparison to general life satisfaction—has changed much more over the 1‐year observation period. In both countries, feelings of depression dropped sharply four months after the lockdown, from 66 percent during the lockdown to 50 percent four months after the lockdown for the South African respondents and from 43 percent to 30 percent for the Ghanaian respondents (see Table 1). However, from August 2020 to March 2021, feelings of depression increased by 5 percentage points in South Africa and 6 percentage points in Ghana, respectively. In general, feelings of depression are much more prevalent in our sample from South Africa than in our sample from Ghana. Interestingly, we find the same pattern when we inspect the results by gender, except that Ghanaian women experienced a constant decrease in feeling depressed, from 50 percent to 38 percent, over time (see Table A5 in the Appendix).

**TABLE 1:** Descriptive Statistics for Key Indicators for South African and Ghanaian Sample

	South Africa					Ghana				
	Mean April 2020	Mean August 2020	Mean March 2021	*p*‐Value Δ April 2020 to August 2020	*p*‐Value Δ April 2020 to March 2021	Mean April 2020	Mean August 2020	Mean March 2021	*p*‐Value Δ April 2020 to August 2020	*p*‐Value Δ April 2020 to March 2021
Outcomes										
Life satisfaction (scale 0–10)	3.9	4.7	5.3	0.036**	0.000***	5.5	5.5	5.3	0.446	0.011**
Feel down, depressed, hopeless (scale 1–5)	2.9	3.4	3.0	0.025**	0.585	3.5	3.9	3.7	0.000***	0.092*
Feel down, depressed, hopeless (binary scale 0–1)	0.66	0.50	0.55	0.009***	0.247	0.43	0.30	0.36	0.001***	0.055*
Economic Factors										
Unemployed or not working (binary scale 0–1)	0.76	0.83	0.71	0.237	0.402	0.19	0.19	0.16	0.852	0.270
Bad financial status (binary scale 0–1)	0.73	0.66	0.59	0.292	0.032**	0.40	0.30	0.25	0.032**	0.000***
Borrowed money (binary scale 0–1)	0.29	0.17	0.31	0.028**	0.653	0.12	0.09	0.10	0.102	0.174
At least once went to bed w/out food within last 7 days (binary scale 0–1)	0.17	0.12	0.11	0.292	0.241	0.06	0.03	0.02	0.001***	0.000***
Food item not available when going grocery shopping (binary scale 0–1)	0.22	0.22	0.12	0.968	0.053*	0.14	0.05	0.04	0.000***	0.000***
Lockdown Stress Factors										
Distrust in government (binary scale 0–1)	0.09	0.42	0.37	0.000***	0.000***	0.18	0.28	0.29	0.000***	0.000***
Never went outside the compound to visit someone in last 7 days (binary scale 0–1)	0.84	0.90	0.66	0.225	0.002***	0.92	0.50	0.34	0.000***	0.000***
Worried about not getting enough food in the near future (binary scale 0–1)	0.90	0.76	0.77	0.008***	0.013**	0.62	0.40	0.46	0.000***	0.000***
Worried about lower future income of my household (binary scale 0–1)	0.96	0.77	0.83	0.000***	0.004***	0.75	0.65	0.79	0.000***	0.101
Afraid of someone at home (binary scale 0–1)	0.17	0.24	0.24	0.209	0.189	0.41	0.24	0.28	0.000***	0.000***
Pandemic Stress Factors										
Bad health condition (binary scale 0–1)	0.06	0.05	0.10	0.648	0.298	0.02	0.03	0.04	0.133	0.046**
Not at least mentioned two COVID‐19 symptoms defined by WHO (binary scale 0–1)	0.46	0.35	0.47	0.100	0.913	0.32	0.44	0.50	0.000***	0.000***
No knowledge of COVID‐19 cases (binary scale 0–1)	0.58	0.91	1.00	0.000***	0.000***	0.48	0.67	0.72	0.000***	0.000***
Worried about health of family (binary scale 0–1)	0.88	0.74	0.81	0.007***	0.159	0.62	0.52	0.66	0.000***	0.079*

*Notes*: Scale for outcome feel down, depressed, hopeless refers to 1 = does not apply at all to 5 = strongly applies. Detailed information about the variables can be found in Tables A4 and A5. All results are reweighted with IPW presented in Table A1. Level of *p*‐values highlighted: * *p* <0.10, ** *p* <0.05, *** *p* <0.01.

Based on the literature on general economic downturns (Avdic et al., 2020; Zajacova et al., 2020; Black et al., 2022), but also specifically on the COVID‐19 shock (Cheng et al., 2020; Mahmud and Riley, 2021; Posel et al., 2021), mental health is expected to be very closely linked to economic development. Unemployment, job insecurity, a bad financial situation, or a lack of liquidity might substantially influence mental health, including life satisfaction and depression.

Figure A3 shows the distribution in working status for all three survey rounds in both countries as well as working status one month before the lockdown, which we elicited from participants in the first survey round (April 2020). Given that all people except essential workers were mandated to stay at home during April 2020 in both Ghana and South Africa most people did not work during the lockdown. However, a substantial share still worked, emphasizing the need for regular income in contexts where social protection schemes are low. Compared to before the lockdown in February 2020, the urban poor in Ghana and South Africa seem to have recovered to a similar working status distribution by March 2021, one year after the lockdown (see Figure A3). Only the share of people not working (i.e., not looking for a job) has increased in South Africa (from 19 percent to 26 percent), whereas the share of unemployed has decreased from 57 percent before the lockdown in February 2020 to 45 percent in March 2021. Figure A3 and Table 1 also indicate that the recovery to pre‐pandemic working status already happened four months after the lockdown in Ghana, whereas we see this recovery only in the third survey round for South Africa. This result is not surprising given that South Africa had a lengthier and stricter social distancing regulations (see Figure 2). Since employment status does not provide the complete picture, i.e., one could be employed, but with a lower income, we also asked respondents if their household income changed between February 2020 (before the lockdown) and February 2021 (almost one year after the lockdown). In South Africa, 66 percent indicated that income stayed the same or even increased and in Ghana, about half reported that their total household income stayed the same or even increased from 2020 to 2021.

Turning to other economic indicators (see Table 1), respondents from the South African sample rate their overall financial situation as being worse than the Ghanaian sample, are more likely to go to bed without food, and are more likely to borrow money. But in both countries, we find a substantial and continuous (from April 2020 to August 2020 to March 2021) reduction in respondents perceiving their financial situation as bad and going to bed hungry. With regard to households' financial liquidity, we find a short relaxation in both countries in August 2020, but liquidity constraints worsened again to April 2020‐levels in March 2021.

To conclude, mental health factors seem to only recover slowly (life satisfaction) or even worsen again (feelings of depression) one year after the lockdown (Table 1), whereas most economic factors seem to have recovered (Table 1) with the easing of governmental restrictions (Figure 2). These results raise the question of what other factors besides economic ones could be driving mental health dynamics over time.

### 4.2 Development of Stress Factors Related to the Global Pandemic and National Lockdowns

In addition to adverse economic impacts, COVID‐19 cases and government measures may have influenced many other factors that have (long‐term) adverse mental health implications. Concerns about personal health, political uncertainty and the related distrust in the government, limited mobility and social isolation, missing knowledge about the pandemic, domestic violence, or anxieties about future incomes might potentially negatively impact mental health, as shown in recent studies for HICs and in previous epidemics (Perrin et al., 2009; Peterman et al., 2020; Pfefferbaum and North, 2020; Banks et al., 2021).

In contrast to economic indicators, which mostly improved between April 2020 and March 2021, we find that most global pandemic stress factors increased over time in both countries (see Table 1). More people rated their personal health to be worse in March 2021 than during the lockdown in 2020. Moreover, in both countries worries about the health of the family, worsened again in March 2021. In Ghana, worries about the health of the family were even larger in March 2021 than in April 2020. Additionally, knowledge about the pandemic has decreased substantially over time in both countries—both related to the total number of COVID‐19 cases in the country (within a 20 percent boundary) and with regard to being able to name at least two of the official WHO symptoms of the disease. Overall, this trend in the lack of knowledge could influence mental health in two different ways. On the one hand, people may no longer care about the pandemic and therefore be less afraid; on the other hand, people may be less informed and therefore more affected in their mental health due to greater uncertainty.

Interestingly, most national lockdown stress factors also worsened between April 2020 and March 2021 even after the lockdowns were gradually lifted. Trust in government and society deteriorated over time. Worries about food and lower income in the near future first decreased between April 2020 and August 2020 but then increased again in March 2021. In contrast and as expected, social isolation decreased over the year. In both countries, where going outside and gathering with other people was prohibited during the lockdown, we find that around 84 percent in the South African sample and 92 percent in the Ghanaian sample did not visit anyone outside of their compound within the last 7 days in April 2020. In Ghana, this rate decreased continuously to 50 percent in August 2020 and 34 percent in March 2021. In South Africa, where strict gathering policies were still in place in August 2020, we only find a substantial decrease in people not visiting others—down to 66 percent in March 2021.

During the lockdown, 41 percent of Ghanaians said they are afraid of someone in their home, but this improved substantially to 24 percent in the second round. The literature supports the sudden improvement after the lockdown in Ghana, as it emphasizes that staying at home and not being able to go outside led to much higher levels of domestic violence worldwide (United Nations, 2020). The level of being afraid of someone at home is higher for women than men during the lockdown (47 percent vs. 38 percent), but results in a similar level after lockdowns were lifted (see Tables A4 and A5 in the Appendix). In contrast, South African respondents were less afraid of someone at home during the lockdown than after (an increase from 17 percent to 24 percent), which could be linked to alcohol sales that were prohibited during the lockdown (first round), but allowed during the second and third rounds. South Africa also saw a decrease in crime rates in spring 2020, especially murder and assaults, partially attributed to the alcohol ban (Burke, 2020).

To conclude, most lockdown and pandemic stress factors have increased over time, except improvements in visiting people outside of own household and fear of domestic violence (for Ghana), as expected to result from the lifting of movement and gathering restrictions.

### 4.3 Factors of COVID‐19 Pandemic Correlated with Life Satisfaction and Depression

In Tables 2 and 3, we bring together the various factors linked to mental health. In Table 2, we use a pooled OLS regression to explore which time‐invariant personal characteristics and time‐variant economic factors may be associated with life satisfaction and feelings of depression across the two cities and across time. Moreover, we compare factors directly linked to the global health crisis with factors linked to national policies restricting social interaction due to the lockdown.

First, and as indicated by Table 1, mental health is significantly worse in South Africa than in Ghana not controlling for other factors (Table 2, columns 1 and 7). Ghana has a statistically significant 0.8 scale point higher life satisfaction (scale from zero to 10) and a 0.6 scale point lower feeling of depression (scale from one to five). Moreover, and again in line with Table 1, we see that life satisfaction did not change much over the three survey rounds and only improved somewhat one year after the first lockdown, whereas feelings of depression improved from the lockdown to August 2020, but interestingly, worsened again to lockdown levels in March 2021 (Table 2, columns 1 and 7).

When only controlling for time‐invariant socio‐economic personal characteristics (Table 2, columns 2 and 8) these country and time effects remain. This is expected for the time effects (with time‐invariant personal characteristics), but shows that observed differences in levels of mental health between South Africa and Ghana (see Table 1) do not seem to be driven by differences in individual characteristics of our samples even though some of those characteristics are indeed correlated with life satisfaction and feelings of depression. We find that tertiary education is positively correlated with life satisfaction, but also the likelihood of feeling depressed. Women are more likely to report that they feel depressed, but no differences between men and women can be found in life satisfaction. The higher reporting of depression by women seems to be linked to stress factors directly linked to health (Table 2, column 11): after controlling for those factors, the gender dummy becomes insignificant. Indicators of household poverty in the sample, i.e., fewer rooms per person and the need to share a toilet or water source, are negatively correlated with life satisfaction but not with feelings of depression. Other personal characteristics either show no or a very weak correlation with life satisfaction or feelings of depression.

The difference in levels of mental health scores between Ghana and South Africa disappears once we control for economic factors (Table 2, columns 3 and 9), such as working conditions and financial status. Therefore, country differences in levels are fully explained by economic factors that are quite different between South Africa and Ghana, in general (see Table 1). Adding economic factors also levels off any changes in life satisfaction across time (Table 2, column 3). Therefore, economic factors, which deteriorated during the lockdowns, seem to play a decisive role in reported life satisfaction between 2020 and 2021.^7^ This is not the case for depression, where an improvement and subsequent deterioration in feelings of depression is observed even when controlling for economic factors (Table 2, column 9).

Therefore, the interesting question is which factors are correlated with changes in feelings of depression over time. Similar to Section 4.2, we first analyze factors that are directly influenced by the health crisis, such as personal health and knowledge about the pandemic, and compare them to factors that are influenced by national restrictions on public life, such as changes in visiting people outside the house or trust in government (Table 2, columns 10 and 11).

**TABLE 2:** Factors Correlating with Life Satisfaction and Feeling Depressed, Pooled OLS Regression

		Life satisfaction (0–10)						Feel down, depressed, hopeless (1–5)				
	(1)	(2)	(3)	(4)	(5)	(6)	(7)	(8)	(9)	(10)	(11)	(12)
Survey round (ref. April 2020)												
August 2020	0.166	0.167	0.0163	−0.103	0.0887	0.0106	−0.403***	−0.402***	−0.263***	−0.0167	−0.243***	−0.0634
	(0.215)	(0.198)	(0.895)	(0.426)	(0.478)	(0.936)	(0.000)	(0.000)	(0.001)	(0.837)	(0.002)	(0.430)
March 2021	0.216*	0.215*	−0.0265	0.0190	0.156	0.196	−0.128	−0.128	0.0603	0.216**	−0.0583	0.0897
	(0.083)	(0.076)	(0.829)	(0.891)	(0.223)	(0.174)	(0.107)	(0.105)	(0.440)	(0.012)	(0.468)	(0.303)
Country (ref. South Africa)												
Ghana	0.827***	1.109***	0.0961	−0.0552	−0.0952	−0.169	−0.613***	−0.762***	−0.125	0.0708	0.0982	0.183
	(0.000)	(0.000)	(0.669)	(0.803)	(0.666)	(0.437)	(0.000)	(0.000)	(0.329)	(0.575)	(0.436)	(0.143)
**Socioeconomic factors**												
Female		−0.141	−0.0428	−0.00572	0.0317	0.0548		0.218***	0.161**	0.110*	0.0852	0.0536
		(0.174)	(0.666)	(0.953)	(0.749)	(0.567)		(0.003)	(0.017)	(0.087)	(0.199)	(0.403)
Age (average in years)		0.0484	0.0708*	0.0516	0.0956**	0.0701*		0.0325	0.0250	0.0322	−0.00342	0.00966
		(0.211)	(0.080)	(0.197)	(0.017)	(0.078)		(0.221)	(0.376)	(0.231)	(0.901)	(0.717)
Household with children (ref. no)		−0.0145	−0.00564	−0.00425	−0.00485	−0.0195		0.0909	0.0721	0.0562	0.0495	0.0518
		(0.887)	(0.955)	(0.964)	(0.960)	(0.836)		(0.192)	(0.274)	(0.368)	(0.443)	(0.403)
Average number of rooms per person		0.453***	0.314***	0.245**	0.290***	0.219**		−0.0898	−0.0118	0.0473	−0.00322	0.0503
		(0.000)	(0.002)	(0.016)	(0.003)	(0.027)		(0.211)	(0.862)	(0.482)	(0.961)	(0.442)
Shared water source with other households		−0.344***	−0.119	−0.201*	−0.0953	−0.174		0.00698	−0.163*	−0.0289	−0.176*	−0.0554
		(0.008)	(0.321)	(0.080)	(0.422)	(0.129)		(0.946)	(0.086)	(0.757)	(0.056)	(0.545)
Shared toilet with other households		−0.385***	−0.199**	−0.0662	−0.132	−0.0317		0.380***	0.235***	0.0899	0.134*	0.0414
		(0.000)	(0.041)	(0.487)	(0.173)	(0.737)		(0.000)	(0.001)	(0.169)	(0.052)	(0.525)
Education (ref. no education)												
Primary education completed only		−0.113	−0.0457	0.0132	−0.0800	−0.0213		0.238*	0.167	0.0777	0.152	0.0767
		(0.584)	(0.809)	(0.943)	(0.669)	(0.907)		(0.085)	(0.197)	(0.526)	(0.234)	(0.528)
Secondary education completed only		0.0334	−0.0584	−0.0701	−0.112	−0.129		0.0339	0.0821	0.0834	0.0984	0.106
		(0.853)	(0.734)	(0.676)	(0.517)	(0.442)		(0.778)	(0.477)	(0.436)	(0.384)	(0.318)
Tertiary education completed		0.832***	0.660***	0.557***	0.580***	0.440**		0.207	0.294*	0.393***	0.267*	0.390***
		(0.000)	(0.002)	(0.006)	(0.008)	(0.033)		(0.189)	(0.058)	(0.007)	(0.076)	(0.006)
**Economic factors**												
Work status (ref. unemployed)												
Self‐employed			0.814***	0.775***	0.808***	0.763***			−0.413***	−0.359***	−0.427***	−0.373***
			(0.000)	(0.000)	(0.000)	(0.000)			(0.001)	(0.004)	(0.001)	(0.003)
Employment without contract			0.0855	0.229	0.138	0.262			0.127	−0.0262	0.0679	−0.0496
			(0.685)	(0.270)	(0.508)	(0.199)			(0.392)	(0.852)	(0.639)	(0.721)
Employment with contract			1.030***	1.009***	1.019***	0.988***			−0.445***	−0.456***	−0.465***	−0.466***
			(0.000)	(0.000)	(0.000)	(0.000)			(0.001)	(0.001)	(0.000)	(0.000)
Not working			0.319	0.228	0.338	0.252			−0.128	−0.0631	−0.153	−0.101
			(0.173)	(0.319)	(0.145)	(0.268)			(0.378)	(0.655)	(0.275)	(0.467)
Bad financial status (ref. good)			−1.024***	−0.759***	−0.960***	−0.712***			0.744***	0.521***	0.656***	0.485***
			(0.000)	(0.000)	(0.000)	(0.000)			(0.000)	(0.000)	(0.000)	(0.000)
Borrowed money (ref. no)			−0.658***	−0.579***	−0.625***	−0.551***			0.465***	0.405***	0.448***	0.398***
			(0.000)	(0.000)	(0.000)	(0.001)			(0.000)	(0.000)	(0.000)	(0.000)
At least once went to bed w/out food within last 7 days			−0.503**	−0.404*	−0.482**	−0.400*			0.311*	0.187	0.247	0.152
			(0.024)	(0.061)	(0.029)	(0.062)			(0.050)	(0.237)	(0.109)	(0.327)
Food item not available when going grocery shopping			−0.132	−0.0727	−0.0709	−0.0616			0.318***	0.223*	0.185	0.152
			(0.459)	(0.663)	(0.691)	(0.713)			(0.009)	(0.058)	(0.122)	(0.196)
**Lockdown stress factors**												
Distrust in government (ref. trust)				−0.124		−0.0961				0.0175		−0.0146
				(0.242)		(0.359)				(0.810)		(0.838)
Never went outside the compound to visit someone in last 7 days				0.160		0.174				0.146**		0.128*
				(0.129)		(0.105)				(0.045)		(0.076)
Worried about not getting enough food in the near future				−0.548***		−0.575***				0.683***		0.600***
				(0.000)		(0.000)				(0.000)		(0.000)
Worried about lower future income of my household				−0.687***		−0.689***				0.414***		0.355***
				(0.000)		(0.000)				(0.000)		(0.000)
Afraid of someone at home				−0.373***		−0.384***				0.663***		0.627***
				(0.001)		(0.001)				(0.000)		(0.000)
**Pandemic stress factors**												
Bad health condition (ref. good)					−0.303	−0.305					0.657***	0.630***
					(0.111)	(0.100)					(0.000)	(0.000)
Not at least mentioned two COVID‐19 symptoms defined by WHO					−0.213**	−0.153					0.0552	0.0197
					(0.024)	(0.101)					(0.373)	(0.747)
No knowledge of COVID‐19 cases					−0.459***	−0.531***					0.149**	0.220***
					(0.000)	(0.000)					(0.028)	(0.001)
Worried about health of family					−0.372***	−0.00130					0.770***	0.411***
					(0.000)	(0.989)					(0.000)	(0.000)
Constant	4.483***	4.003***	4.827***	5.766***	5.413***	6.149***	3.074***	2.702***	2.000***	0.862***	1.470***	0.659***
	(0.000)	(0.000)	(0.000)	(0.000)	(0.000)	(0.000)	(0.000)	(0.000)	(0.000)	(0.000)	(0.000)	(0.004)
Observations	2480	2480	2479	2476	2470	2470	2485	2485	2484	2481	2475	2475
Adjusted *R*‐squared	0.031	0.081	0.190	0.242	0.205	0.254	0.043	0.061	0.162	0.295	0.221	0.317

*Notes*: Pooled OLS regression of life satisfaction and depression. Scale for outcome feel down, depressed, hopeless refers to 1 = does not apply at all to 5 = strongly applies. Detailed information about the variables can be found in Tables A4 and A5. Results are robust with an ordered logit model and are presented in Table A7 in the Appendix. All results are reweighted with IPW presented in Table A1. *p*‐Values in parentheses: * *p* <0.10, ** *p* <0.05, *** *p* <0.01. Models have fewer observations if people said they do not want to answer question about life satisfaction or depression.

The predictive power of our model (adjusted *R* ^2^) to explain the variance in feelings of depression across individuals and time changes somewhat more if we control for factors related to the lockdown rather than factors related to the pandemic (Table 2, columns 10 and 11; 0.30 vs. 0.22). Factors related to the pandemic and the lockdown together seem to be driving changes in feelings of depression over time (Table 2, column 12). If we only control for factors related to the lockdown, i.e., distrust in government, lack of social interaction, being afraid of somebody at home, and worries about future income and food, feelings of depression even increase over time. In other words, when controlling for differences in factors induced by governmental restrictions on social interaction, feelings of depression even increased over time, which might be linked to the length of the pandemic and increasing uncertainty about it (which unfortunately we cannot test with our data).

The results of the panel specification with individual fixed effects (Table 3) are very similar to the pooled OLS regression (Table 2), highlighting that there is little unobserved time‐invariant heterogeneity across individuals that we did not control for in Table 2. The downside of a panel is that we cannot analyze the effects of personal social characteristics and country fixed effects, as these are captured by the individual fixed effects. The advantage is that the panel allows us to control for time‐invariant individual heterogeneity with a fixed effects model, i.e., that certain respondents might in general be more prone to mental health issues due to unobservable characteristics. There is one main difference between the panel (Table 3) and the pooled regression (Table 2). If, in addition to economic factors, we control for the various stress factors, life satisfaction actually improves over time (Table 3, column 3), and does not stay constant (Table 2, column 6). Therefore, stress factors seem to be also driving constant life satisfaction over time in addition to feelings of depression.

With regard to individual stress factors, worse knowledge about the pandemic seems to be highly correlated with worse mental health (Table 3, columns 3 and 6). Therefore, being informed leads to better mental health or better mental health leads to information‐seeking behavior. Moreover, worries about future income, food security and family health are also highly correlated with both mental health indicators. Trust in the government does not seem to play a role. Low mobility is only negatively correlated with life satisfaction and being afraid of somebody at home only with feelings of depression (Table 3, columns 3 and 6). In Table 3, we also run the panel regressions separately for Ghana and South Africa to analyze whether coefficients are different across the countries (the cross‐sectional OLS regressions for each individual country can be found in Table A6 in the Appendix). We note the following differences between Ghana and South Africa. Life satisfaction of the urban poor in Ghana is highly correlated with economic factors such as job security, the financial situation, and food security, as well as worries about future income and trust in the government. In contrast, in our South African sample, mental health is less correlated with economic factors and not at all correlated with trust in government, but more so with poor current physical health.

**TABLE 3:** Factors Correlating with Life Satisfaction and Feeling Depressed, Fixed Effect Panel Regression

	Life satisfaction (0 to +10)			Feel down, depressed, hopeless (1–5)		
	South Africa	Ghana	Both	South Africa	Ghana	Both
	(1)	(2)	(3)	(4)	(5)	(6)
Time Fixed Effect (ref. April 2020)						
August 2020	0.792*	−0.165*	0.156	−0.261	−0.0406	−0.168**
	(0.059)	(0.087)	(0.231)	(0.248)	(0.611)	(0.037)
March 2021	1.895***	−0.179*	0.414***	−0.224	0.157*	0.0168
	(0.000)	(0.066)	(0.003)	(0.296)	(0.073)	(0.849)
**Economic factors**						
Work status (ref. unemployed)						
Self‐employed	0.767	0.605**	0.899**	−0.288	−0.453**	−0.382*
	(0.524)	(0.014)	(0.012)	(0.627)	(0.028)	(0.054)
Employment without contract	0.231	0.584**	0.935***	0.386	−0.105	0.000409
	(0.643)	(0.029)	(0.002)	(0.265)	(0.642)	(0.998)
Employment with contract	1.576***	1.007***	1.294***	−0.425	−0.499**	−0.430**
	(0.008)	(0.000)	(0.000)	(0.244)	(0.018)	(0.033)
Not working	−0.392	0.0804	0.278	0.386	−0.0341	0.184
	(0.539)	(0.778)	(0.472)	(0.301)	(0.890)	(0.427)
Bad financial status (ref. good)	−0.920**	−0.840***	−0.779***	0.244	0.490***	0.431***
	(0.017)	(0.000)	(0.000)	(0.337)	(0.000)	(0.000)
Borrowed money (ref. no)	−1.528***	0.0777	−0.564***	0.702***	0.168	0.415***
	(0.000)	(0.617)	(0.004)	(0.002)	(0.143)	(0.000)
At least once went to bed w/out food within last 7 days	−0.394	−0.272	−0.532*	0.0159	0.237	0.157
	(0.440)	(0.198)	(0.071)	(0.956)	(0.154)	(0.338)
Food item not available when going grocery shopping	0.947***	−0.488***	0.172	−0.353	0.462***	0.0590
	(0.010)	(0.006)	(0.404)	(0.141)	(0.002)	(0.668)
**Lockdown stress factors**						
Distrust in government (ref. trust)	0.408	−0.277***	−0.0100	−0.156	0.141*	0.0505
	(0.292)	(0.007)	(0.940)	(0.529)	(0.079)	(0.578)
Never went outside the compound to visit someone in last 7 days	−0.0925	0.209**	0.402***	−0.0707	0.125	0.0637
	(0.793)	(0.026)	(0.001)	(0.758)	(0.115)	(0.405)
Worried about not getting enough food in the near future	−1.528***	−0.311***	−0.450***	0.219	0.503***	0.440***
	(0.000)	(0.004)	(0.000)	(0.397)	(0.000)	(0.000)
Worried about lower future income of my household	−0.0618	−0.598***	−0.575***	0.488	0.336***	0.410***
	(0.887)	(0.000)	(0.000)	(0.134)	(0.000)	(0.000)
Afraid of someone at home	−0.406	−0.0532	−0.0710	0.119	0.485***	0.380***
	(0.234)	(0.595)	(0.585)	(0.616)	(0.000)	(0.000)
**Pandemic stress factor**						
Bad health condition (ref. good)	−1.436***	−0.148	−0.562**	0.855**	0.567***	0.691***
	(0.002)	(0.570)	(0.017)	(0.041)	(0.003)	(0.000)
Not at least mentioned two COVID‐19 symptoms defined by WHO	−0.0864	−0.0742	−0.128	−0.139	−0.0332	−0.0325
	(0.775)	(0.380)	(0.235)	(0.453)	(0.631)	(0.642)
No knowledge of COVID‐19 cases	−1.778***	−0.566***	−0.728***	0.513	0.180**	0.217***
	(0.000)	(0.000)	(0.000)	(0.108)	(0.013)	(0.007)
Worried about health of family	−0.369	−0.307***	−0.373***	0.637**	0.104	0.238**
	(0.350)	(0.003)	(0.002)	(0.022)	(0.205)	(0.012)
Constant	7.748***	6.386***	6.012***	1.254**	1.424***	1.372***
	(0.000)	(0.000)	(0.000)	(0.033)	(0.000)	(0.000)
Observations	363	2107	2470	361	2114	2475
Adjusted *R*‐squared	0.341	0.218	0.195	0.146	0.235	0.195

*Notes*: Fixed effect panel regression of life satisfaction and depression. Scale for outcome feel down, depressed, hopeless refers to 1 = does not apply at all to 5 = strongly applies. Detailed information about the variables can be found in Tables A4 and A5. Results are robust with an ordered logit model and are presented in Table A9 in the Appendix. All results are reweighted with IPW presented in Table A1. *p*‐Values in parentheses: * *p* <0.10, ** *p* <0.05, *** *p* <0.01. Models have fewer number of observations if people said they do not want to answer question about life satisfaction or depression.

## 5 Discussion and Conclusion

COVID‐19 has had a significant impact on the world economy, especially on those individuals who are living in poverty. As many LMICs implemented strict government measures to reduce social interaction early in the COVID‐19 pandemic to slow down the spreading of the virus, many people, and especially the urban poor, were as affected by lockdown policies as by the disease itself. Although the global pandemic was and is still ongoing, most LMICs have relaxed social distancing regulations a couple of months after the first COVID‐19 cases around the world were detected.

The end of public lockdowns with a global health crisis ongoing at the same time raises questions about how people's well‐being has changed as they start to re‐enter a new public life—especially in terms of their mental health, which has barely been studied after lockdowns in LMICs. The status of mental health recovery even after public lockdowns ended is an essential question, since experts warned that due to the changed social environment, continued high levels of uncertainty, and lasting economic downturns (Zajacova et al., 2020), we should expect a “tsunami” of mental illness post‐pandemic (Royal College of Psychiatrists, 2020). To this end, we analyze how mental health in two middle‐income countries with strict lockdowns has evolved from the start of the pandemic to one year after, and ask which factors are associated with changes in mental health and observed differences across countries.

In this study, we focus on one of the most vulnerable population groups: households living in the poorest settlements in major cities in LMIC countries. This group has a high share of people working in the informal economy, low incomes, poor housing conditions, and limited social security and savings. Thus, our results are not generalizable to the entire country, however, they contribute to the few studies focusing on recovery in poor communities (Bishi et al., 2020; IPA, 2020; Porter et al., 2021; Rönkkö et al., 2021; World Bank, 2021). Our results could help policy makers assist vulnerable communities transition out of severe public movement and social interaction restrictions, knowledge that may also be needed for future pandemics.

The longitudinal study analyzes over 1,000 poor urban households living in Greater Johannesburg area, South Africa, and Greater Accra area, Ghana, over the span of one year. The two LMICs countries had some of the highest COVID‐19 case numbers in Africa, a similar timing of peaks in cases, and a similar start to their COVID‐19 vaccine campaigns, but had different governmental measures in place with regard to stringency and length. Moreover, the economic situation of their urban poor also differs substantially, with many South African urban poor unemployed and many Ghanaian urban poor self‐employed.

As most government regulations were relaxed one year into the pandemic, the economic indicators that dropped considerably during lockdown mostly recovered one year after the lockdown both in urban Ghana and South Africa. Therefore, the lockdown and pandemic seemed to have little long‐term economic effects on the urban poor. Ghana has recovered faster, which is expected, given that Ghana only implemented a short lockdown.

Since economic downturns have previously been shown to be strongly associated with a deterioration in mental health, one might assume that mental health factors also fell during the lockdown and should have recovered faster in Ghana than in South Africa over one year. Analyzing self‐reported life satisfaction and the feeling of being depressed, we find that respondents' life satisfaction in South Africa was relatively low at the beginning of the lockdown, but improved slightly though gradually over the subsequent year, whereas in Ghana, life satisfaction stayed constant (but at a higher level than in South Africa). On the contrary, for feeling depressed, urban poor households in Johannesburg and Accra both reported a short‐term recovery in August 2020, but experienced increasing feelings of depression again in March 2021. Most previous studies on mental health in LMICs are cross‐sectional and were conducted only when strict social distancing measures were in place (Shamoon et al., 2020; Cénat et al., 2021; Goularte et al., 2021; Wang et al., 2021). Therefore, at least to our knowledge, we are the first to analyze mental health dynamics in LMICs up to one year after the start of the pandemic. Moreover, these results indicate that the correlation between economic and mental well‐being recovery might be lower than expected.

In addition to economic impacts, COVID‐19 and the measures to slow the virus's reproduction have influenced many other factors that might have adverse mental health implications. We find that most stress factors related to a global pandemic and national lockdowns (such as physical health, worries about the health of family members, trust in government, and knowledge about the pandemic, worries about future income) have actually increased over time, except visiting people outside of one's own household and the fear of domestic violence (for Ghana), which was expected due to lifted movement and gathering policies.

This study is also one of the first in LMICs to extensively analyze the correlation of two mental health outcomes with economic factors as well as factors directly linked to a global health crisis and factors linked to public movement restrictions. The results contribute to the longitudinal studies (Cheng et al., 2020; Zajacova et al., 2020; Banks et al., 2021; Matsubayashi et al., 2022) for HICs. Our results indicate that differences in economic factors explain the differences in mental health between South Africa and Ghana and are indeed highly correlated with mental health outcomes. The ongoing mental health crisis (despite substantial improvements in economic indicators) is, according to our results, mainly linked to stress factors related to both the global pandemic and national lockdowns. In particular, differences in mental health are correlated with varying knowledge about the pandemic, physical health, and worries about the future rather than lack of mobility. Nevertheless, important country differences exist: economic factors and trust in the government seem to be more important in Ghana, whereas health factors seem to be more important in South Africa.

This study has a number of limitations. First, we do not have any pre‐pandemic indicators except working status, which we obtained during the first round in April 2020 for February 2020. Moreover, the sampling strategy was different for the two countries. However, we show that differences in individual characteristics across the two samples do not seem to drive our results. Third, having to rely on a phone survey during the pandemic resulted in high attrition rates, in particular for the South African sample, which we addressed by inverse probability weighting and limiting our observations to a balanced sample.

Our results indicate that we need to consider a broader range of needs when designing policies to accelerate post‐pandemic recovery in the well‐being of the urban poor—and that these factors might vary across countries. Moreover, supportive mental health policies could be offered, especially to poor households, as mental health is not only linked to economic factors, but anxieties linked to the pandemic and the associated governmental measures. Additionally, the government should try to increase knowledge about the pandemic among the population.

## Supporting information


**Figure A.1:** Map of study area in South Africa
**Figure A.2:** Map of study area in Ghana
**Figure A.3:** Working status in South Africa and Ghana
**Figure A.4:** Working status in South Africa and Ghana, by gender
**Table A.1:** Attrition in the sample of South Africa and Ghana
**Table A.2:** Descriptive Statistics Of Key Characteristics Of South Africa and Ghana Sample Over the Three Survey Rounds
**Table A.3:** Correlation Matrix of Mental Health Factors and Anxieties Indicators in South Africa and Ghana
**Table A.4:** Descriptive Statistics for Key Indicators for South African and Ghanaian Sample, Male
**Table A.5:** Descriptive statistics for key indicators for south African and Ghanaian sample, female
**Table A.6:** Factors Correlating with Life Satisfaction and Feeling Depressed, by Gender and Pooled OLS Regression
**Table A.7:** Factors Correlating with the Feeling Depressed, Pooled Ordered Logit Regression
**Table A.8:** Factors Correlating with Life Satisfaction and Feeling Depressed, Fixed Effect Panel Regression by Gender
**Table A.9:** Factors Correlating with Feeling Depressed, Fixed Effect Ordered Logit

## Footnotes

1 Note that testing rates are different between HICs and LMICs, potentially influencing the number of confirmed cases.

2 Abeka, Ablekuma, Accra New Town, Alajo, Ashaiman, Chorkor, Gbegbeyise, Jamestown, Kokomlemle, Kotobabi, La, Maamobi, Madina, Mamprobi, Nima, Pig Farm, Sabon Zongo, Ussher Town.

3 Etwatwa and Daveyton

4 In both countries we had already ongoing research collaborations with universities.

5 All suburbs where the local partner conducted their project on low‐smoke stoves were selected. A series of random coordinates were chosen per suburb. The street blocks in which each randomly chosen coordinate fell were selected and all households in these street blocks were interviewed.

6 As Table A3 in the Appendix shows, these worries are not highly correlated with each other. Thus, we do not assume that worries are just a representation of mental health, but that they identify specific worries in people's life.

7 Note that we do not know whether in 2021 life satisfactions were back to pre‐pandemic levels.
