# Supplementary material for: Economic Recovery but Stagnating Mental Health During a Global Pandemic? Evidence from Ghana and South Africa
Source: Rev Income Wealth. 2022 May 2;68(2):563–89. doi: 10.1111/roiw.12587 (PMC9348047; doi:10.1111/roiw.12587)
Supplement: Supplementary file 1 — Figure A.1: Map of study area in South Africa Figure A.2: Map of study area in Ghana Figure A.3: Working status in South Africa and Ghana Figure A.4: Working status in South Africa and Ghana, by gender Table A.1: Attrition in the sample of South Africa and Ghana Table A.2: Descriptive Statistics Of Key Characteristics Of South Africa and Ghana Sample Over the Three Survey Rounds Table A.3: Correlation Matrix of Mental Health Factors and Anxieties Indicators in South Africa and Ghana Table A.4: Descriptive Statistics for Key Indicators for South African and Ghanaian Sample, Male Table A.5: Descriptive statistics for key indicators for south African and Ghanaian sample, female Table A.6: Factors Correlating with Life Satisfaction and Feeling Depressed, by Gender and Pooled OLS Regression Table A.7: Factors Correlating with the Feeling Depressed, Pooled Ordered Logit Regression Table A.8: Factors Correlating with Life Satisfaction and Feeling Depressed, Fixed Effect Panel Regression by Gender Table A.9: Factors Correlating with Feeling Depressed, Fixed Effect Ordered Logit [file ROIW-68-563-s001.pdf]

Appendix

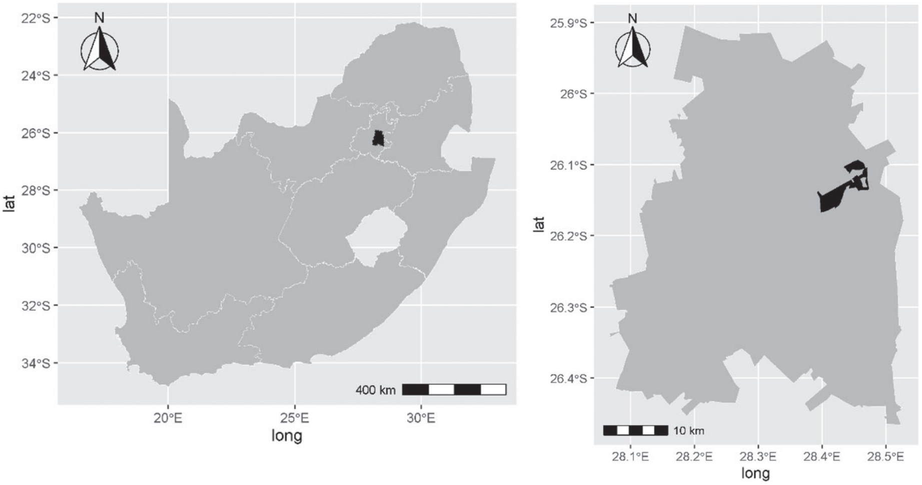

Figure A1. Map of study area in South Africa. The left panel in the graph above shows all nine provinces of South Africa, black highlighted the municipality of Ekurhuleni in the province Gauteng where the two neighborhoods are located. In the right panel, the two neighborhoods of Ekurhuleni, Etwatwa and Daveyton, are highlighted in black

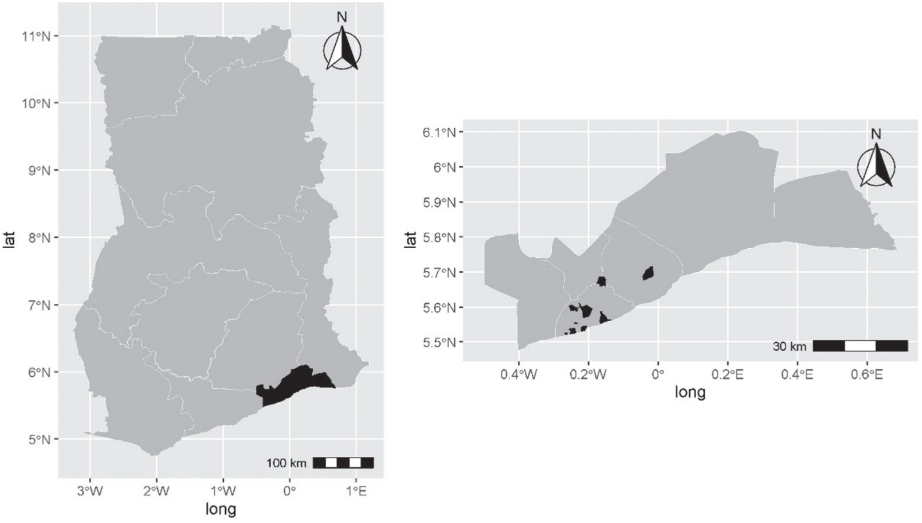

Figure A2. Map of study area in Ghana. The left panel in the graph above shows all 10 regions of Ghana, black highlighted the region Greater Accra where the 18 neighborhoods are located. Ghana reorganized the 10 regions into 16 regions in 2019. Since the official shape files are not yet available and the boundary stayed the same for Greater Accra, the old boundaries are shown in the graph. In the right panel the region Greater Accra with the six districts are shown. In black 16 neighborhoods are highlighted. Pig Farm and Ablekuma are not yet included since the boundaries were not yet confirmed by the statistical department

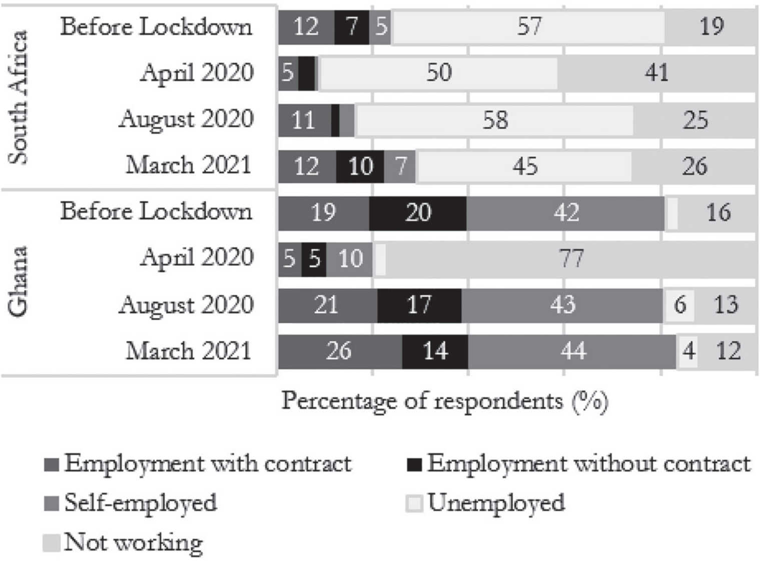

Figure A3. Working status in South Africa and Ghana. Based on question "What is your main working status?" The category "Not working" refers to the categories: Unable to work due to disability, not looking for a job, student, retired person, and homemaker/housewife. During the lockdown, in April 2020, all respondents who reported that they cannot work/business is closed due to the lockdown were additionally categorized as "Not working." Results are reweighted with IPW presented in Table A1. In South Africa the change from April 2020, August 2020, March 2021 relative to before lockdown for employment with contract are −0.07 ($p = 0.071$), −0.01 ($p = 0.842$), 0.00 ($p = 0.946$), respectively. Employment without contract −0.04 ($p = 0.160$), −0.06 ($p = 0.054$), 0.03 ($p = 0.473$). Self-employed −0.04 ($p = 0.115$), −0.02 ($p = 0.568$), 0.02 ($p = 0.530$). Unemployed −0.07 ($p = 0.288$), 0.00 ($p = 0.931$), −0.12 ($p = 0.072$). Not working 0.22 ($p = 0.000$), 0.06 ($p = 0.235$), 0.07 ($p = 0.196$). In Ghana the change from April 2020, August 2020, March 2021 relative to before lockdown for employment with contract are −0.14 ($p = 0.000$), 0.02 ($p = 0.472$), 0.07 ($p = 0.003$), respectively. Employment without contract −0.15 ($p = 0.000$), −0.03 ($p = 0.201$), −0.06 ($p = 0.003$). Self-employed −0.32 ($p = 0.000$), 0.01 ($p = 0.700$), 0.02 ($p = 0.435$). Unemployed −0.00 ($p = 0.903$), 0.03 ($p = 0.002$), 0.02 ($p = 0.100$). Not working 0.61 ($p = 0.000$), −0.03 ($p = 0.103$), −0.04 ($p = 0.031$)

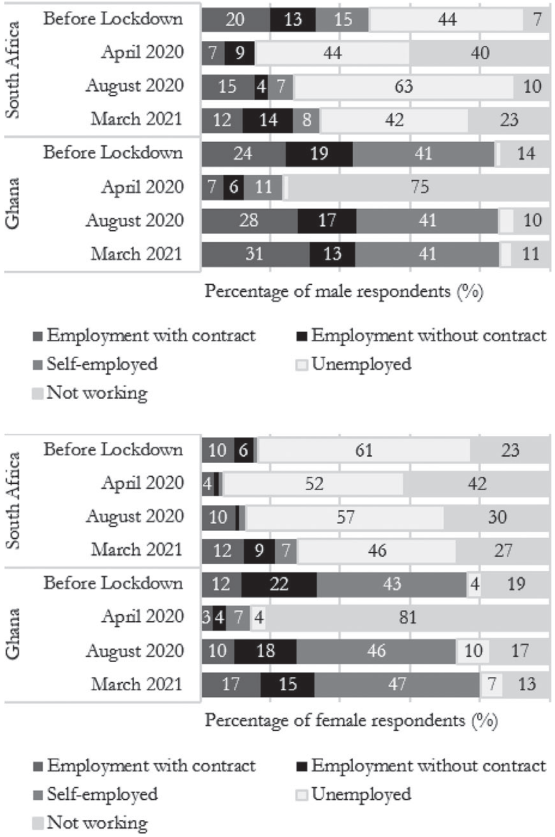

Figure A4. Working status in South Africa and Ghana, by gender. Based on question "What is your main working status?" The category "Not working" refers to the categories: Unable to work due to disability, not looking for a job, student, retired person, and homemaker /housewife. During the lockdown, in April 2020, all respondents were additionally categorized as "Not working" who reported that they cannot work/ business is closed due to the lockdown. Results are reweighted with IPW presented in Table A1. Left panel refers to male respondents: In South Africa the change from April 2020, August 2020, March 2021 relative to before lockdown for employment with contract are −0.13 ($p = 0.183$), −0.05 ($p = 0.647$), −0.08 ($p = 0.452$), respectively. Employment without contract −0.05 ($p = 0.581$), −0.09 ($p = 0.221$), 0.01 ($p = 0.907$). Self-employed −0.15 ($p = 0.038$), −0.08 ($p = 0.349$), −0.08 ($p = 0.400$). Unemployed −0.00 ($p = 1.000$), 0.17 ($p = 0.205$), −0.02 ($p = 0.891$). Not working 0.33 ($p = 0.002$), 0.03 ($p = 0.678$), 0.16 ($p = 0.071$). In Ghana the change from April 2020, August 2020, March 2021 relative to before lockdown for employment with contract are −0.17 ($p = 0.000$), 0.03 ($p = 0.250$), 0.07 ($p = 0.022$), respectively. Employment without contract −0.13 ($p = 0.000$), −0.02 ($p = 0.438$), −0.06 ($p = 0.027$). Self-employed −0.29 ($p = 0.000$), 0.00 ($p = 0.979$), 0.00 ($p = 0.831$). Unemployed −0.00 ($p = 835$), 0.02 ($p = 0.034$), 0.01 ($p = 0.203$). Not working 0.61 ($p = 0.000$), −0.04 ($p = 0.110$), −0.03 ($p = 0.213$). The right panel refers to female respondents: In South Africa the change from April 2020, August 2020, March 2021 relative to before lockdown for employment with contract are −0.06 ($p = 0.202$), 0.00 ($p = 0.947$), 0.03 ($p = 0.597$), respectively. Employment without contract −0.04 ($p = 0.155$), −0.04 ($p = 0.135$), 0.03 ($p = 0.417$). Self-employed 0.00 ($p = 1.000$), 0.00 ($p = 0.743$), 0.05 ($p = 0.089$). Unemployed −0.09 ($p = 0.223$), −0.05 ($p = 0.543$), −0.15 ($p = 0.047$). Not working 0.19 ($p = 0.007$), 0.07 ($p = 0.258$), 0.04 ($p = 0.538$). In Ghana the change from April 2020, August 2020, March 2021 relative to before lockdown for employment with contract are −0.08 ($p = 0.001$), −0.02 ($p = 0.469$), 0.06 ($p = 0.067$), respectively. Employment without contract −0.18 ($p = 0.000$), −0.04 ($p = 0.288$), −0.07 ($p = 0.058$). Self-employed −0.36 ($p = 0.000$), 0.03 ($p = 0.542$), 0.05 ($p = 0.312$). Unemployed −0.00 ($p = 1.000$), 0.05 ($p = 0.020$), 0.02 ($p = 0.252$). Not working 0.62 ($p = 0.000$), −0.02 ($p = 0.538$), −0.06 ($p = 0.070$).

TABLE A1

Attrition in the sample of South Africa and Ghana

	South Africa		Ghana	
	Attrition 1st to 2nd survey round	Attrition 1st to 3rd survey round	Attrition 1st to 2nd survey round	Attrition 1st to 3rd survey round
Female	1.007	0.960	1.341	1.507***
	(0.982)	(0.891)	(0.123)	(0.009)
Age (average in years)	0.744**	0.809*	1.048	1.061
	(0.010)	(0.064)	(0.545)	(0.360)
Household members	0.934	0.877	0.962	0.974
	(0.446)	(0.108)	(0.363)	(0.440)
Number of rooms	1.069	1.064	1.007	0.946
	(0.525)	(0.536)	(0.925)	(0.381)
Average number of rooms per person	0.809	0.657	1.126	1.532*
	(0.519)	(0.146)	(0.671)	(0.064)
Shared water source with other households	2.891*	2.680	0.838	1.119
	(0.053)	(0.144)	(0.526)	(0.596)
Shared toilet with other households	0.998	0.379**	0.831	1.050
	(0.997)	(0.039)	(0.364)	(0.773)
Education (ref. no education)				
Primary education completed only-	1.351	1.031	1.393	1.021
	(0.400)	(0.923)	(0.464)	(0.954)
Secondary education completed only	1.894	1.292	1.586	1.187
	(0.119)	(0.512)	(0.200)	(0.531)
Tertiary education completed	1.908	1.640	1.417	1.469
	(0.258)	(0.425)	(0.440)	(0.268)

(continued)

TABLE A1
(Continued)

	South Africa		Ghana	
	Attrition 1st to 2nd survey round	Attrition 1st to 3rd survey round	Attrition 1st to 2nd survey round	Attrition 1st to 3rd survey round
Working status before lockdown (ref. unemployed)				
Self-employed	0.410	0.940	0.508	1.006
	(0.227)	(0.926)	(0.215)	(0.989)
Employed without contract	1.129	0.787	0.389*	0.637
	(0.813)	(0.630)	(0.100)	(0.349)
Employed with contract	0.918	1.178	1.233	1.088
	(0.856)	(0.743)	(0.743)	(0.876)
Not working	2.903***	1.216	1.348	1.368
	(0.004)	(0.594)	(0.573)	(0.497)
Main source of income for the household (before lockdown)				
Salary from work	1.230	1.194	0.494	0.813
	(0.606)	(0.669)	(0.133)	(0.579)
Own business	1.846	1.335	1.325	1.052
	(0.350)	(0.651)	(0.337)	(0.832)
National grants	1.316	0.965	0.135***	0.447*
	(0.367)	(0.908)	(0.003)	(0.063)
Support from family members	1.009	1.054	0.865	0.859
	(0.983)	(0.897)	(0.572)	(0.463)
Other source of income	0.906	0.688	0.448**	0.705
	(0.861)	(0.474)	(0.043)	(0.224)
Constant	0.609	3.289	0.175	0.272
	(0.569)	(0.150)	(0.015)	(0.027)
Observation	394	394	998	998
R-squared	0.062	0.042	0.044	0.031

Note: Logit regression of the binary variable if person left between first and second, first and third survey round, respectively. Odds ratios are shown with p-values in parentheses: *p <0.10, **p <0.05, ***p <0.01. To account for the attrition of the first to the third waves, Inverse Probability Weighting (IPW) was applied to the results.

TABLE A2

DESCRIPTIVE STATISTICS OF KEY CHARACTERISTICS OF SOUTH AFRICA AND GHANA SAMPLE OVER THE THREE SURVEY ROUNDS

	South Africa			Ghana		
	1st round $n = 409$	2nd round $n = 274$	3rd round $n = 128$	1st round $n = 1{,}034$	2nd round $n = 863$	3rd round $n = 736$
Female (%)	75	74	76	37	36	35
Age (average in years)	40–49	40–49	40–49	40–49	40–49	40–49
Household members	5.2	5.2	5.2	5.3	5.4	5.5
Number of rooms	4.5	4.4	4.5	2.4	2.3	2.4
Average number of rooms per person	1.0	1.0	1.1	0.6	0.6	0.6
Shared water source with other households (%)	6	3	2	13	14	13
Shared toilet with other households (%)	8	6	9	66	67	67
Education						
No education level completed (%)	20	23	25	8	9	8
Primary education completed only (%)	44	46	47	9	9	9
Secondary education completed only (%)	30	25	23	68	68	69
Tertiary education completed (%)	7	6	5	15	15	14
Working status (before lockdown)						
Unemployed (%)	57	59	58	4	4	4
Self-employed (%)	4	5	4	40	40	39
Employed without contract (%)	6	6	7	21	22	23
Employed with contract (%)	12	12	9	20	20	20
Not working (%)	21	18	23	15	14	14
Main sources of income for the household (before lockdown)						
Salary from work (%)	22	20	17	23	23	22
Own business (%)	5	6	5	58	58	59
National grants (%)	64	62	65	5	5	5
Support from family members (%)	11	11	11	20	20	20
Other source of income (%)	7	7	8	9	9	9

Note: Number of household members was specified up to 11 people—more than 11 are counted as 12 for the average calculation. Number of rooms was specified up to 10 rooms—more than 10 are counted as 11 for the average calculation. Working status and main income source refers to the baseline response to understand if there was a systematic attrition. Not working status includes housewife/homemaker, retired person/pensioner, school pupil/full-time student, unable to work due to disability, and unemployed not looking for a job. For the household's main sources of income, the respondents could mention several sources. National grants include child support grants, old-age pensions, and disability grants.

TABLE A3

Correlation Matrix of Mental Health Factors and Anxieties Indicators in South Africa and Ghana

Number of followed measures		Life satisfaction	Feeling down, depressed, hopeless	Worried about health family	Worried no food	Worried no income
South Africa	Life satisfaction	1.0000				
	Feeling down, depressed, hopeless	−0.3815	1.0000			
	Worried about health family	−0.1568	0.2280	1.0000		
	Worried no food	−0.2892	0.2769	0.3285	1.0000	
	Worried no income	−0.1660	0.2211	0.2263	0.4698	1.0000
Ghana	Life satisfaction	1.0000				
	Feeling down, depressed, hopeless	−0.3668	1.0000			
	Worried about health family	−0.1166	0.3479	1.0000		
	Worried no food	−0.3294	0.5110	0.4491	1.0000	
	Worried no income	−0.4044	0.4442	0.3584	0.5212	1.0000

Note: The variables feeling down, depressed, hopeless; Worried about health of family; Worried about no food; Worried about no income were asked on a five-scale from strongly agree to strongly disagree. Results are reweighted with IPW presented in Table A1.

TABLE A4

Descriptive Statistics for Key Indicators for South African and Ghanaian Sample, Male

Male	South Africa Mean April 2020	South Africa Mean August 2020	South Africa Mean March 2021	South Africa *p*-Value Δ April 2020 to August 2020	South Africa *p*-Value Δ April 2020 to March 2021	Ghana Mean April 2020	Ghana Mean August 2020	Ghana Mean March 2021	Ghana *p*-Value Δ April 2020 to August 2020	Ghana *p*-Value Δ April 2020 to March 2021
Outcomes										
Life satisfaction (scale 0–10)	3.7	4.6	5.2	0.199	0.031**	5.8	5.7	5.3	0.610	0.001***
Feel down, depressed, hopeless (scale 1–5)	2.7	3.5	3.0	0.064*	0.559	3.7	4.1	3.7	0.000***	0.699
Feel down, depressed, hopeless (binary scale 0–1)	0.64	0.46	0.52	0.111	0.578	0.39	0.26	0.34	0.002***	0.432
Economic Factors										
Unemployed or not working (binary scale 0–1)	0.52	0.71	0.66	0.140	0.286	0.15	0.14	0.14	0.820	0.729
Bad financial status (binary scale 0–1)	0.63	0.65	0.59	0.894	0.724	0.38	0.26	0.24	0.000***	0.000***
Borrowed money (binary scale 0–1)	0.23	0.28	0.37	0.666	0.263	0.12	0.09	0.11	0.128	0.573
At least once went to bed w/out food within last 7 days (binary scale 0–1)	0.15	0.26	0.13	0.275	0.864	0.05	0.02	0.02	0.004***	0.009***
Food item not available when going grocery shopping (binary scale 0–1)	0.31	0.11	0.25	0.077*	0.647	0.13	0.04	0.04	0.000***	0.000***
Lockdown Stress Factors										
Distrust in government (binary scale 0–1)	0.12	0.38	0.47	0.033**	0.005***	0.18	0.29	0.30	0.000***	0.000***
Never went outside the compound to visit someone in last 7 days (binary scale 0–1)	0.91	0.85	0.47	0.527	0.000***	0.92	0.48	0.35	0.000***	0.000***
Worried about not getting enough food in the near future (binary scale 0–1)	0.89	0.67	0.74	0.045**	0.147	0.57	0.39	0.46	0.000***	0.001***
Worried about lower future income of my household (binary scale 0–1)	0.96	0.83	0.81	0.092*	0.077*	0.70	0.63	0.77	0.018**	0.011**
Afraid of someone at home (binary scale 0–1)	0.21	0.29	0.14	0.435	0.505	0.38	0.23	0.27	0.000***	0.000***
Pandemic Stress Factors										
Bad Health Condition (binary scale 0–1)	0.05	0.02	0.12	0.528	0.309	0.01	0.02	0.03	0.152	0.009***
Not at least mentioned two COVID-19 symptoms defined by WHO (binary scale 0–1)	0.52	0.44	0.37	0.521	0.251	0.33	0.42	0.47	0.004***	0.000***
No knowledge of COVID-19 cases (binary scale 0–1)	0.55	0.89	1.00	0.003***	0.000***	0.41	0.60	0.68	0.000***	0.000***
Worried about health of family (binary scale 0–1)	0.80	0.73	0.83	0.529	0.786	0.59	0.48	0.64	0.001***	0.090*

Note: Based on questions “On a scale from 0 to 10, if 0 is not at all satisfied and 10 is completely satisfied. How satisfied are you with your life at the moment?”, “Can you tell me how much this statement applies to you? I feel down, depressed, hopeless” (1 = does not apply at all to 5 = strongly applies), “What is your main working status?” (displayed as binary if unemployed or not working, April 2020 refers to before lockdown, a detailed figure can be found in Appendix A3), “How would you rate the overall financial condition of your household today?” (displayed as binary if bad or very bad), “Did you borrow money last week?” (displayed as binary if yes), “How many days did you go to bed without food during the last week?” (displayed as binary if at least once), “The last time you went shopping, were there any items you wanted to buy but were not available?” (displayed as binary if yes), “How much do you trust the government to take care of the people?” (displayed as binary if strongly distrust or somewhat distrust), “How many people have you visited outside of your house in the last 7 days?” (displayed as binary if never), “I am worried about not getting enough food in the near future” (displayed as binary if does strongly apply or somewhat apply), “I am worried about lower future income of my household in the future” (displayed as binary if does strongly apply or somewhat apply), “I am afraid of someone I am sharing the house with” (displayed as binary if does strongly apply or somewhat apply), “How would you consider your health?” (displayed as binary if terrible or bad), “What are the symptoms of coronavirus?” (displayed as binary if not more than two official WHO symptoms are mentioned), “How many people do you think have been infected by the coronavirus?” (displayed as binary if number not within 20% boundary of correct cases), “I am worried about the health of my family (displayed as binary if does strongly apply or somewhat apply). Results are reweighted with IPW presented in Table A1 Level of *p*-values highlighted: * $p<0.10$, ** $p<0.05$, *** $p<0.01$.

TABLE A5

DESCRIPTIVE STATISTICS FOR KEY INDICATORS FOR SOUTH AFRICAN AND GHANAIAN SAMPLE, FEMALE

Female	South Africa Mean April 2020	South Africa Mean August 2020	South Africa Mean March 2021	South Africa p-Value Δ April 2020 to August 2020	South Africa p-Value Δ April 2020 to March 2021	Ghana Mean April 2020	Ghana Mean August 2020	Ghana Mean March 2021	Ghana p-Value Δ April 2020 to August 2020	Ghana p-Value Δ April 2020 to March 2021
Outcomes										
Life satisfaction (scale 0–10)	3.9	4.7	5.3	0.091*	0.001***	5.2	5.1	5.2	0.547	0.792
Feel down, depressed, hopeless (scale 1–5)	3.0	3.3	3.0	0.138	0.778	3.3	3.6	3.6	0.031**	0.029**
Feel down, depressed, hopeless (binary scale 0–1)	0.67	0.52	0.56	0.038**	0.312	0.50	0.38	0.39	0.134	0.040**
Economic Factors										
Unemployed or not working (binary scale 0–1)	0.84	0.86	0.72	0.655	0.093*	0.25	0.27	0.20	0.625	0.218
Bad financial status (binary scale 0–1)	0.76	0.66	0.59	0.197	0.024**	0.43	0.36	0.29	0.124	0.001***
Borrowed money (binary scale 0–1)	0.31	0.13	0.30	0.005***	0.905	0.13	0.11	0.09	0.462	0.141
At least once went to bed w/out food within last 7 days (binary scale 0–1)	0.17	0.07	0.11	0.041**	0.214	0.08	0.04	0.02	0.076*	0.001***
Food item not available when going grocery shopping (binary scale 0–1)	0.19	0.25	0.08	0.361	0.039**	0.16	0.06	0.03	0.000***	0.000***
Lockdown Stress Factors										
Distrust in government (binary scale 0–1)	0.08	0.44	0.33	0.000***	0.000***	0.18	0.28	0.27	0.009***	0.020**
Never went outside the compound to visit someone in last 7 days (binary scale 0–1)	0.82	0.91	0.72	0.084*	0.149	0.93	0.54	0.33	0.000***	0.000***
Worried about not getting enough food in the near future (binary scale 0–1)	0.90	0.79	0.78	0.062*	0.044**	0.69	0.42	0.47	0.000***	0.000***
Worried about lower future income of my household (binary scale 0–1)	0.95	0.75	0.84	0.000***	0.022**	0.83	0.69	0.81	0.000***	0.468
Afraid of someone at home (binary scale 0–1)	0.16	0.22	0.27	0.323	0.069*	0.47	0.26	0.29	0.000***	0.000***
Pandemic Stress Factors										
Bad health condition (binary scale 0–1)	0.06	0.06	0.09	0.818	0.552	0.04	0.06	0.06	0.359	0.578
Not at least mentioned two COVID-19 symptoms defined by WHO (binary scale 0–1)	0.44	0.33	0.50	0.126	0.451	0.31	0.47	0.57	0.000***	0.000***
No knowledge of COVID-19 cases (binary scale 0–1)	0.58	0.91	1.00	0.000***	0.000***	0.60	0.80	0.79	0.000***	0.000***
Worried about health of family (binary scale 0–1)	0.91	0.74	0.81	0.006***	0.068*	0.67	0.58	0.70	0.047**	0.493

Note: Based on questions "On a scale from 0 to 10, if 0 is not at all satisfied and 10 is completely satisfied. How satisfied are you with your life at the moment?", "Can you tell me how much this statement applies to you? I feel down, depressed, hopeless" (1 = does not apply at all to 5 = strongly applies), "What is your main working status?" (displayed as binary if unemployed or not working, April 2020 refers to before lockdown, a detailed figure can be found in Figure A3), "How would you rate the overall financial condition of your household today?" (displayed as binary if bad or very bad), "Did you borrow money last week?" (displayed as binary if yes), "How many days did you go to bed without food during the last week?" (displayed as binary if at least once), "The last time you went shopping, were there any items you wanted to buy but were not available?" (displayed as binary if yes), "How much do you trust the government to take care of the people?" (displayed as binary if strongly distrust or somewhat distrust), "How many people have you visited outside of your house in the last 7 days?" (displayed as binary if never), "I am worried about not getting enough food in the near future" (displayed as binary if does strongly apply or somewhat apply), "I am worried about lower future income of my household in the future" (displayed as binary if does strongly apply or somewhat apply), "I am afraid of someone I am sharing the house with" (displayed as binary if does strongly apply or somewhat apply), "How would you consider your health?" (displayed as binary if terrible or bad), "What are the symptoms of coronavirus?" (displayed as binary if not more than two official WHO symptoms are mentioned), "How many people do you think have been infected by the coronavirus?" (displayed as binary if number not within 20% boundary of correct cases), "I am worried about the health of my family (displayed as binary if does strongly apply or somewhat apply). Results are reweighted with IPW presented in Table A1 Level of p-values highlighted: * p <0.10, ** p <0.05, *** p <0.01.

TABLE A6

Factors Correlating with Life Satisfaction and Feeling Depressed, by Gender and Pooled OLS Regression

	Life satisfaction (0–10)					Feel down, depressed, hopeless (1–5)				
	South Africa (1)	Ghana (2)	Both (3)	Both Male (4)	Both Female (5)	South Africa (6)	Ghana (7)	Both (8)	Both Male (9)	Both Female (10)
Survey round (ref. April 2020)										
August 2020	0.689*	−0.244**	0.0106	−0.0917	0.0388	−0.273	0.0685	−0.0634	−0.121	0.0132
	(0.089)	(0.019)	(0.936)	(0.526)	(0.855)	(0.238)	(0.367)	(0.430)	(0.236)	(0.916)
March 2021	1.534***	−0.317***	0.196	−0.230	0.597**	−0.103	0.195**	0.0897	0.148	0.0357
	(0.000)	(0.003)	(0.174)	(0.131)	(0.011)	(0.639)	(0.021)	(0.303)	(0.176)	(0.788)
Country (ref. South Africa)										
Ghana			−0.169	0.0981	−0.378			0.183	0.0316	0.233
			(0.437)	(0.773)	(0.167)			(0.143)	(0.886)	(0.151)
Socioeconomic factors										
Female	0.441	−0.0447	0.0548			−0.00875	0.0476	0.0536		
	(0.133)	(0.556)	(0.567)			(0.965)	(0.414)	(0.403)		
Age (average in years)	0.165	0.0591*	0.0701*	0.0777*	0.0649	−0.0776	0.0341	0.00966	−0.00533	0.0267
	(0.185)	(0.063)	(0.078)	(0.080)	(0.300)	(0.330)	(0.156)	(0.717)	(0.874)	(0.516)
Household with children (ref. no)	0.237	0.146	0.219**	0.112	0.522***	0.175	−0.0629	0.0503	0.0133	0.0243
	(0.224)	(0.131)	(0.027)	(0.329)	(0.003)	(0.174)	(0.321)	(0.442)	(0.868)	(0.838)
Average number of rooms per person	−0.0165	−0.0771	−0.0195	−0.0918	0.0495	0.0882	0.0895	0.0518	0.102	0.0130
	(0.952)	(0.312)	(0.836)	(0.385)	(0.746)	(0.644)	(0.112)	(0.403)	(0.157)	(0.899)
Shared water source with other households	−0.149	−0.229**	−0.174	−0.337**	−0.0201	−0.459	−0.0184	−0.0554	−0.0720	−0.0328
	(0.769)	(0.029)	(0.129)	(0.019)	(0.911)	(0.311)	(0.827)	(0.545)	(0.485)	(0.847)
Shared toilet with other households	0.702	−0.182**	−0.0317	−0.139	0.176	−0.0379	−0.00247	0.0414	0.0325	0.0594
	(0.146)	(0.034)	(0.737)	(0.216)	(0.289)	(0.900)	(0.969)	(0.525)	(0.680)	(0.602)
Education (ref. no education)										
Primary education completed only	0.101	−0.211	−0.0213	0.190	−0.0930	−0.0671	0.0492	0.0767	0.188	0.103
	(0.769)	(0.204)	(0.907)	(0.618)	(0.662)	(0.751)	(0.715)	(0.528)	(0.378)	(0.493)
Secondary education completed only	−0.560	−0.0937	−0.129	0.0109	−0.169	0.0480	−0.0104	0.106	0.316*	0.0514
	(0.234)	(0.513)	(0.442)	(0.972)	(0.409)	(0.855)	(0.925)	(0.318)	(0.064)	(0.709)
Tertiary education completed	0.712	0.195	0.440**	0.386	0.653*	0.756*	0.195	0.390***	0.480**	0.715***
	(0.202)	(0.284)	(0.033)	(0.252)	(0.067)	(0.094)	(0.147)	(0.006)	(0.012)	(0.007)
Economic factors										
Work status (ref. unemployed)										
Self-employed	0.550	0.867***	0.763***	0.900***	0.871***	−0.136	−0.385***	−0.373***	−0.597***	−0.227
	(0.414)	(0.000)	(0.000)	(0.002)	(0.001)	(0.669)	(0.010)	(0.003)	(0.002)	(0.154)
Employment without contract	−0.0790	0.350*	0.262	0.444	0.236	0.577	−0.180	−0.0496	−0.215	0.00406
	(0.861)	(0.064)	(0.199)	(0.126)	(0.364)	(0.130)	(0.248)	(0.721)	(0.301)	(0.983)
Employment with contract	1.112**	1.132***	0.988***	1.265***	0.796**	−0.398	−0.455***	−0.466***	−0.629***	−0.435**
	(0.040)	(0.000)	(0.000)	(0.000)	(0.044)	(0.126)	(0.003)	(0.000)	(0.002)	(0.019)
Not working	−0.166	0.404**	0.252	0.486	0.159	−0.147	−0.0175	−0.101	−0.353	0.00104
	(0.669)	(0.050)	(0.268)	(0.160)	(0.565)	(0.513)	(0.916)	(0.467)	(0.109)	(0.995)

(continued)

TABLE A6
(CONTINUED)

	Life satisfaction (0–10)					Feel down, depressed, hopeless (1–5)				
	South Africa (1)	Ghana (2)	Both (3)	Both Male (4)	Both Female (5)	South Africa (6)	Ghana (7)	Both (8)	Both Male (9)	Both Female (10)
Bad financial status (ref. good)	−0.681**	−0.820***	−0.712***	−0.900***	−0.562***	0.571***	0.473***	0.485***	0.538***	0.449***
	(0.034)	(0.000)	(0.000)	(0.000)	(0.003)	(0.002)	(0.000)	(0.000)	(0.000)	(0.000)
Borrowed money (ref. no)	−1.401***	0.0486	−0.551***	0.116	−1.105***	0.633***	0.254**	0.398***	0.198	0.579***
	(0.000)	(0.720)	(0.001)	(0.546)	(0.000)	(0.001)	(0.017)	(0.000)	(0.146)	(0.000)
At least once went to bed w/out food within last 7 days	−0.299	−0.160	−0.400*	−0.427	−0.288	0.196	0.272	0.152	0.228	0.0891
	(0.427)	(0.443)	(0.062)	(0.146)	(0.343)	(0.428)	(0.108)	(0.327)	(0.301)	(0.672)
Food item not available when going grocery shopping	0.266	−0.369**	−0.0616	−0.324	0.187	−0.370	0.507***	0.152	0.367**	−0.0347
	(0.415)	(0.014)	(0.713)	(0.144)	(0.418)	(0.105)	(0.000)	(0.196)	(0.010)	(0.842)
Lockdown stress factors										
Distrust in government (ref. trust)	0.449	−0.296***	−0.0961	−0.200*	0.0185	0.168	−0.0470	−0.0146	−0.0786	0.0596
	(0.189)	(0.000)	(0.359)	(0.097)	(0.916)	(0.408)	(0.478)	(0.838)	(0.356)	(0.603)
Never went outside the compound to visit someone in last 7 days	−0.621*	0.124	0.174	0.0575	0.299*	0.322	0.121*	0.128*	0.121	0.119
	(0.065)	(0.170)	(0.105)	(0.623)	(0.093)	(0.155)	(0.077)	(0.076)	(0.171)	(0.307)
Worried about not getting enough food in the near future	−1.012**	−0.559***	−0.575***	−0.559***	−0.540***	0.401	0.636***	0.600***	0.523***	0.663***
	(0.010)	(0.000)	(0.000)	(0.000)	(0.006)	(0.106)	(0.000)	(0.000)	(0.000)	(0.000)
Worried about lower future income of my household	−0.505	−0.643***	−0.689***	−0.626***	−0.697***	0.264	0.344***	0.355***	0.330***	0.385***
	(0.193)	(0.000)	(0.000)	(0.000)	(0.001)	(0.307)	(0.000)	(0.000)	(0.000)	(0.004)
Afraid of someone at home	−0.764**	−0.303***	−0.384***	−0.516***	−0.218	0.284	0.737***	0.627***	0.706***	0.510***
	(0.025)	(0.001)	(0.001)	(0.000)	(0.237)	(0.157)	(0.000)	(0.000)	(0.000)	(0.000)
Pandemic stress factors										
Bad health condition (ref. good)	−0.940**	−0.000456	−0.305	−0.368	−0.233	0.552*	0.628***	0.630***	0.734***	0.551***
	(0.011)	(0.998)	(0.100)	(0.268)	(0.319)	(0.078)	(0.001)	(0.000)	(0.006)	(0.009)
Not at least mentioned two COVID-19 symptoms defined by WHO	0.204	−0.221***	−0.153	−0.227**	−0.0828	−0.0547	0.0141	0.0197	0.0352	−0.00913
	(0.453)	(0.002)	(0.101)	(0.027)	(0.585)	(0.738)	(0.803)	(0.747)	(0.632)	(0.926)
No knowledge of COVID-19 cases	−1.167***	−0.438***	−0.531***	−0.353***	−0.715***	0.236	0.228***	0.220***	0.163**	0.286**
	(0.005)	(0.000)	(0.000)	(0.000)	(0.000)	(0.373)	(0.000)	(0.001)	(0.021)	(0.022)
Worried about health of family	−0.215	0.126	−0.00130	0.128	−0.219	0.470**	0.364***	0.411***	0.316***	0.559***
	(0.530)	(0.109)	(0.989)	(0.192)	(0.231)	(0.033)	(0.000)	(0.000)	(0.000)	(0.000)
Constant	6.499***	6.296***	6.149***	5.914***	5.927***	1.042	0.859***	0.659***	0.977***	0.413
	(0.000)	(0.000)	(0.000)	(0.000)	(0.000)	(0.110)	(0.000)	(0.004)	(0.005)	(0.205)
Observations	363	2107	2470	1470	1000	361	2114	2475	1476	999
Adjusted *R*-squared	0.247	0.295	0.254	0.294	0.222	0.143	0.364	0.317	0.334	0.281

Note: OLS regression of life satisfaction and depression by country and gender and pooled. Scale for outcome feeling down, depressed, hopeless refers to 1 = does not apply at all to 5 = strongly applies. Detailed information about the variables can be found in Tables A4 and A5. All results are reweighted with IPW presented in Table A1. p-Values in parentheses: * $p<0.10$, ** $p<0.05$, *** $p<0.01$. Models have fewer observations if people said they do not want to answer question about life satisfaction or depression.

TABLE A7

Factors Correlating with the Feeling Depressed, Pooled Ordered Logit Regression

	Feel down, depressed, hopeless (1–5)			
	(1)	(2)	(3)	(4)
Survey round (ref. April 2020)				
August 2020	0.610***	0.605***	0.710***	0.962
	(0.000)	(0.000)	(0.003)	(0.762)
March 2021	0.998	0.996	1.309***	1.414***
	(0.983)	(0.967)	(0.008)	(0.007)
Country (ref. South Africa)				
Ghana	0.493***	0.406***	0.903	1.469**
	(0.000)	(0.000)	(0.526)	(0.031)
**Socioeconomic factors**				
Female		1.330***	1.318***	1.185*
		(0.001)	(0.002)	(0.087)
Age (average in years)		1.047	1.050	1.036
		(0.168)	(0.198)	(0.402)
Average number of rooms per person		0.915	0.993	1.112
		(0.329)	(0.936)	(0.311)
Household with children (ref. no)		1.124	1.136	1.113
		(0.176)	(0.151)	(0.273)
Shared water source with other households		1.011	0.807*	0.896
		(0.929)	(0.099)	(0.426)
Shared toilet with other households		1.641***	1.386***	1.084
		(0.000)	(0.001)	(0.440)
Education (ref. no education)				
Primary education completed only		1.380*	1.303	1.171
		(0.050)	(0.109)	(0.363)
Secondary education completed only		1.054	1.139	1.225
		(0.719)	(0.386)	(0.195)
Tertiary education completed		1.325	1.555**	2.067***
		(0.147)	(0.030)	(0.001)
**Economic factors**				
Work status (ref. unemployed)			0.591***	0.573***
Self-employed			(0.001)	(0.001)
			1.238	0.973
Employment without contract			(0.238)	(0.885)
			0.578***	0.498***
Employment with contract			(0.001)	(0.000)
			0.846	0.887
Not working			(0.354)	(0.541)
			2.681***	2.057***

(continued)

TABLE A7
(Continued)

	Feel down, depressed, hopeless (1–5)			
	(1)	(2)	(3)	(4)
Bad financial status (ref. good)			(0.000)	(0.000)
			1.757***	1.700***
Borrowed money (ref. no)			(0.000)	(0.000)
			1.526**	1.289
At least once went to bed w/out food within last 7 days			(0.034)	(0.248)
			1.606***	1.315
Food item not available when going grocery shopping			(0.003)	(0.118)
			0.591***	0.573***
**Lockdown stress factors**				
Distrust in government (ref. trust)				1.019
				(0.869)
Never went outside the compound to visit someone in last 7 days				1.373***
				(0.004)
Worried about not getting enough food in the near future				2.465***
				(0.000)
Worried about lower future income of my household				2.146***
				(0.000)
Afraid of someone at home				2.396***
				(0.000)
**Pandemic stress factors**				
Bad health condition (ref. good)				2.425***
				(0.000)
Not at least mentioned two COVID-19 symptoms defined by WHO				1.037
				(0.699)
No knowledge of COVID-19 cases				1.399***
				(0.001)
Worried about health of family				2.155***
				(0.000)
Observations	2485	2485	2484	2475
Pseudo R-squared	0.016	0.024	0.064	0.141

Note: Ordered logit model of depression. Odds ratios larger than 1 mean an increased feeling of down, depressed, hopeless. Scale for outcome feel down, depressed, hopeless refers to 1 = does not apply at all to 5 = strongly applies. Detailed information about the variables can be found in Tables A4 and A5. All results are reweighted with IPW presented in Table A1. p-Values in parentheses: * p <0.10, ** p <0.05, *** p <0.01. Models have a smaller number of observations if people said they do not want to answer question about life satisfaction or depression.

TABLE A8
Factors Correlating with Life Satisfaction and Feeling Depressed, Fixed Effect Panel Regression by Gender

	Life satisfaction (0 to +10)		Feel down, depressed, hopeless (1–5)			
	Male (1)	Female (2)	Both (3)	Male (4)	Female (5)	Both (6)
Time Fixed Effect (ref. April 2020)						
August 2020	0.120	0.0681	0.156	−0.238**	−0.0473	−0.168**
	(0.408)	(0.736)	(0.231)	(0.040)	(0.673)	(0.037)
March 2021	0.0722	0.652***	0.414***	0.0824	−0.0391	0.0168
	(0.643)	(0.002)	(0.003)	(0.474)	(0.775)	(0.849)
**Economic Factors**						
Work status (ref. unemployed)						
Self-employed	0.566	1.309***	0.899**	−0.767**	−0.204	−0.382*
	(0.360)	(0.001)	(0.012)	(0.011)	(0.411)	(0.054)
Employment without contract	0.780	1.094***	0.935***	−0.279	0.0877	0.000409
	(0.168)	(0.002)	(0.002)	(0.311)	(0.748)	(0.998)
Employment with contract	1.112**	1.586***	1.294***	−0.771***	−0.246	−0.430**
	(0.046)	(0.000)	(0.000)	(0.007)	(0.419)	(0.033)
Not working	0.844	−0.0505	0.278	−0.187	0.301	0.184
	(0.228)	(0.912)	(0.472)	(0.562)	(0.284)	(0.427)
Bad financial status (ref. good)	−0.728***	−0.784***	−0.779***	0.579***	0.287*	0.431***
	(0.000)	(0.000)	(0.000)	(0.000)	(0.053)	(0.000)
Borrowed money (ref. no)	0.149	−1.209***	−0.564***	0.204	0.583***	0.415***
	(0.526)	(0.000)	(0.004)	(0.145)	(0.000)	(0.000)
At least once went to bed w/out food within last 7 days	−0.271	−0.753*	−0.532*	0.148	0.182	0.157
	(0.417)	(0.084)	(0.071)	(0.518)	(0.412)	(0.338)
Food item not available when going grocery shopping	−0.354	0.540*	0.172	0.314**	−0.0912	0.0590
	(0.160)	(0.055)	(0.404)	(0.042)	(0.659)	(0.668)
**Lockdown Stress Factors**						
Distrust in government (ref. trust)	−0.164	0.220	−0.0100	0.115	−0.0380	0.0505
	(0.281)	(0.299)	(0.940)	(0.323)	(0.778)	(0.578)

(continued)

TABLE A8
(Continued)

	Life satisfaction (0 to +10)		Feel down, depressed, hopeless (1–5)			
	Male (1)	Female (2)	Both (3)	Male (4)	Female (5)	Both (6)
Never went outside the compound to visit someone in last 7 days	0.279*	0.537***	0.402***	0.0575	0.0176	0.0637
	(0.054)	(0.004)	(0.001)	(0.577)	(0.881)	(0.405)
Worried about not getting enough food in the near future	−0.316**	−0.556***	−0.450***	0.302**	0.575***	0.440***
	(0.022)	(0.008)	(0.000)	(0.010)	(0.000)	(0.000)
Worried about lower future income of my household	−0.557***	−0.551**	−0.575***	0.278***	0.610***	0.410***
	(0.000)	(0.013)	(0.000)	(0.009)	(0.000)	(0.000)
Afraid of someone at home	−0.190	−0.0233	−0.0710	0.412***	0.347***	0.380***
	(0.208)	(0.905)	(0.585)	(0.000)	(0.008)	(0.000)
**Pandemic Stress Factor**						
Bad health condition (ref. good)	−0.519	−0.520*	−0.562**	0.716**	0.689***	0.691***
	(0.274)	(0.067)	(0.017)	(0.016)	(0.005)	(0.000)
Not at least mentioned two COVID-19 symptoms defined by WHO	−0.174	−0.0432	−0.128	0.0136	−0.0723	−0.0325
	(0.145)	(0.804)	(0.235)	(0.881)	(0.514)	(0.642)
No knowledge of COVID-19 cases	−0.542***	−0.964***	−0.728***	0.0741	0.377***	0.217***
	(0.000)	(0.000)	(0.000)	(0.423)	(0.008)	(0.007)
Worried about health of family	−0.230*	−0.533**	−0.373***	0.235**	0.266	0.238**
	(0.083)	(0.021)	(0.002)	(0.026)	(0.113)	(0.012)
Constant	5.974***	6.215***	6.012***	1.866***	0.979***	1.372***
	(0.000)	(0.000)	(0.000)	(0.000)	(0.001)	(0.000)
Observations	1470	1000	2470	1476	999	2475
Adjusted R-squared	0.167	0.269	0.195	0.205	0.199	0.195

Note: Fixed effect panel regression of life satisfaction and depression. Scale for outcome feel down, depressed, hopeless refers to 1 = does not apply at all to 5 = strongly applies. Detailed information about the variables can be found in Tables A4 and A5. All results are reweighted with IPW presented in Table A1. p-Values in parentheses: * p <0.10, ** p <0.05, *** p <0.01. Models have fewer number of observations if people said they do not want to answer question about life satisfaction or depression.

TABLE A9

FACTORS CORRELATING WITH FEELING DEPRESSED, FIXED EFFECT ORDERED LOGIT

	Feel down, depressed, hopeless (1–5)		
	South Africa (1)	Ghana (2)	Both (3)
Time Fixed Effect (ref. April 2020)			
August 2020	0.619	0.933	0.729**
	(0.161)	(0.668)	(0.024)
March 2021	0.665	1.277	0.975
	(0.206)	(0.149)	(0.864)
**Economic Factors**			
Work Status (ref. Unemployed)			
Self-employed	0.800	0.397**	0.517*
	(0.776)	(0.020)	(0.053)
Employment without contract	1.750	0.807	0.984
	(0.312)	(0.610)	(0.959)
Employment with contract	0.519	0.423**	0.487**
	(0.241)	(0.035)	(0.039)
Not working	1.857	1.283	1.716
	(0.230)	(0.579)	(0.166)
Bad financial status (ref. good)	1.390	2.323***	1.945***
	(0.340)	(0.000)	(0.000)
Borrowed money (ref. no)	3.175***	1.421*	2.215***
	(0.000)	(0.092)	(0.000)
At least once went to bed w/out food within last 7 days	0.941	1.387	1.138
	(0.888)	(0.232)	(0.625)
Food item not available when going grocery shopping	0.558	1.889**	1.013
	(0.118)	(0.012)	(0.951)
**Lockdown Stress Factors**			
Distrust in government (ref. trust)	0.815	1.324*	1.091
	(0.575)	(0.089)	(0.596)
Never went outside the compound to visit someone in last 7 days	0.773	1.301*	1.125
	(0.493)	(0.099)	(0.411)
Worried about not getting enough food in the near future	1.792	2.340***	2.108***
	(0.121)	(0.000)	(0.000)
Worried about lower future income of my household	1.903	1.970***	2.230***
	(0.206)	(0.002)	(0.000)
Afraid of someone at home	1.360	1.911***	1.678***
	(0.413)	(0.000)	(0.001)
**Pandemic Stress Factors**			
Bad health condition (ref. good)	2.948*	5.499***	3.855***
	(0.070)	(0.000)	(0.000)
Not at least mentioned two COVID-19 symptoms defined by WHO	0.731	0.956	0.909
	(0.303)	(0.745)	(0.458)
No knowledge of COVID-19 cases	2.164	1.299*	1.439**
	(0.107)	(0.070)	(0.013)
Worried about health of family	2.333**	1.438*	1.661***
	(0.026)	(0.051)	(0.004)
Observations	323	1591	1914
Pseudo R-squared	0.214	0.288	0.242

Note: Fixed effect ordered logit model of depression. Odds ratios larger than 1 mean an increased feeling of down, depressed, hopeless. Scale for outcome feel down, depressed, hopeless refers to 1 = does not apply at all to 5 = strongly applies. Detailed information about the variables can be found in Tables A4 and A5. All results are reweighted with IPW presented in Table A1. p-Values in parentheses: * $p < 0.10$, ** $p < 0.05$, *** $p < 0.01$. Models have a smaller number of observations if people said they do not want to answer question about life satisfaction or depression.
